# Voice analytics in the wild: Validity and predictive accuracy of common audio-recording devices

**DOI:** 10.3758/s13428-023-02139-9

**Published:** 2023-05-30

**Authors:** Francesc Busquet, Fotis Efthymiou, Christian Hildebrand

**Affiliations:** Institute of Behavioral Science and Technology, University of St. Gallen, Torstrasse 25, St. Gallen, 9000 Switzerland

**Keywords:** Audio-recording devices, Crowdsourcing, Audio data, Voice analytics, Amplitude, Fundamental frequency

## Abstract

The use of voice recordings in both research and industry practice has increased dramatically in recent years—from diagnosing a COVID-19 infection based on patients’ self-recorded voice samples to predicting customer emotions during a service center call. Crowdsourced audio data collection in participants’ natural environment using their own recording device has opened up new avenues for researchers and practitioners to conduct research at scale across a broad range of disciplines. The current research examines whether fundamental properties of the human voice are reliably and validly captured through common consumer-grade audio-recording devices in current medical, behavioral science, business, and computer science research. Specifically, this work provides evidence from a tightly controlled laboratory experiment analyzing 1800 voice samples and subsequent simulations that recording devices with high proximity to a speaker (such as a headset or a lavalier microphone) lead to inflated measures of amplitude compared to a benchmark studio-quality microphone while recording devices with lower proximity to a speaker (such as a laptop or a smartphone in front of the speaker) systematically reduce measures of amplitude and can lead to biased measures of the speaker’s true fundamental frequency. We further demonstrate through simulation studies that these differences can lead to biased and ultimately invalid conclusions in, for example, an emotion detection task. Finally, we outline a set of recording guidelines to ensure reliable and valid voice recordings and offer initial evidence for a machine-learning approach to bias correction in the case of distorted speech signals.

**Supplementary Information:**

The online version contains supplementary material available at 10.3758/s13428-023-02139-9.

## Introduction

Researchers across disciplines have leveraged the unique insights from unobtrusively collecting and analyzing vocal features from the human voice. Analyzing features in the human voice provides a unique and rich source of information to assess, for example, affective states of individuals (Bachorowski & Owren, 1995; Johnstone & Scherer, 2000; Scherer, 2003), personality traits (Scherer, 1978), assess the attractiveness of individuals (Borkowska & Pawlowski, 2011; Zuckerman & Driver, 1989), predict their physiological predispositions (Pisanski et al., 2016a, b), assess early onset of physical health issues (Kelchner et al., 2010), mental disorders (Faurholt-Jepsen et al., 2016), early diagnosis of the Parkinson’s disease (Brabenec et al., 2017; Erdogdu Sakar et al., 2017), or a COVID-19 infection (Brown et al., 2020; Han et al., 2021).

A large majority of prior research used crowdsourced recruiting methods such as Prolific, Amazon Mechanical Turk, and large-scale recruitment via social media platforms or dedicated mobile apps. This paradigm shift in data collection helped to recruit more participants faster and at a lower cost. However, the key question the current paper asks is whether such voice recordings provide truly reliable and valid measures of the human voice. The key objective of this paper is to provide a causal test to assess whether the use of a broad range of common (and previously used) audio-recording devices allows researchers to reliably and validly measure key vocal features in the human voice.

In what follows, we first provide a short review of prior research on crowdsourced audio data collection, the potential impact of environmental as well as device-related effects on vocal measurements, followed by a causal experiment to assess the reliability and validity of two fundamental measures of the human voice (fundamental frequency and amplitude). Finally, we discuss the implications of the current findings, raise awareness on how unexpected device effects can lead to invalid conclusions, and provide a set of guidelines to ensure reliable and valid voice recordings.

## Literature review

### Crowdsourced audio data collection and variation in recording devices

An increasing number of researchers and practitioners leverage crowdsourcing online platforms (e.g., Amazon Mechanical Turk and Prolific), social media platforms (e.g., Facebook and Twitter), mobile apps, and websites to collect audio data from respondents. Crowdsourced online data collection has numerous benefits: It is efficient, convenient, relatively cheap, and a fast way to gather large amounts of data from diverse populations (Ilieva et al., 2002; Lefever et al., 2007).

However, crowdsourced online data collection comes with important challenges and limitations. Unlike tightly controlled lab experiments, crowdsourcing online data can be influenced by environmental factors (Crump et al., 2013; Palan & Schitter, 2018) as well as equipment variability and require technical knowledge to properly use the equipment (Lefever et al., 2007). In the case of reliably capturing audio data, various factors can influence the captured vocal signal, its analysis, and the inferred conclusions. Such factors include environmental conditions (e.g., background noise) (Lefever et al., 2007), placement of the audio-recording device relative to the source (Mubeen et al., 2012), and even the audio-recording device itself (Krik et al., 2019; Parsa & Jamieson, 2001). Prior work on the role of different commercial devices in capturing reliable audio data has focused to a large extent on voice recordings via smartphone devices for clinical purposes and with limited voice samples (see Table 1). An emerging body of medical research used different audio-recording devices for tasks such as screening laryngeal (Uloza et al., 2015) and lung (Li et al., 2017) diseases or even diagnosing COVID-19 from voice data (Han et al., 2021) (see Table 1 for an overview and summary).

**Table 1:** Summary of prior work utilizing different audio-recording devices

Authors	Audio-recording setting	Audio-recording devices	Conditions	Analyzed vocal features	Feature extraction & analysis program	Key findings
Manfredi et al., 2017	Recordings of 18 synthesized voice samples in a soundproof booth. Smartphones were fixed at a 10 cm distance from the center of the sound source (loudspeaker). The voice samples consisted of sustained /a:/ utterances, of 2 s long, at two different median f0 (120 Hz, 200 Hz), three levels of jitter (0.9%, 2.8%, 4.5%), and three levels of additive noise (97.4 dB, 23.8 dB, 17.6 dB)	Smartphones, external microphone	A basic, inexpensive smartphone (Wiko model CINK SLIM2), a high-level, expensive smartphone (HTC One), and a high-quality external microphone (Sennheiser model MD421U)	Jitter, shimmer, noise-to-harmonics ratio	PRAAT	The results obtained with the three devices for the different jitter, shimmer, and amount of noise levels were significantly correlated. No absolute differences between devices were tested or reported
Guidi et al., 2015	Two subjects were asked to read non-emotional text and to comment on displayed pictures, in the context of an Android application testing, while getting recorded simultaneously by two identical smartphones and one external microphone. The first smartphone was kept on the table and the other was held by the subject. The smartphone held by the subject and the external microphone were positioned at a 30 cm distance from the subjects’ mouths, whereas the smartphone on the table was placed at a 40 cm distance from the subjects’ mouths. In a second experiment, they simultaneously recorded with two different smartphones, both held close together by the subject (the exact distance is not specified)	Smartphones, external microphone	Two Samsung smartphones (I9300 Galaxy S III), an LG smartphone (Nexus 4 E960), and a high-quality external microphone (AKG P220)	Mean f0, standard deviation of f0, and jitter	C code in the Java Native Interface	For both subjects, the three investigated features between the external microphone and hand-held smartphone, external microphone and smartphone on the table, and the two identical smartphones showed significant correlations. Weaker correlations, but still significant, were found for the extracted jitter between the external microphone and the hand-held smartphone. High correlations were also found in all features between the two different smartphones. No differences between devices were tested or reported
Uloza et al., 2015	118 subjects (34 healthy (23 females & 11 males; *M*age = 41.8 years, *SD*age = 16.96) and 84 pathological voices of various voice disorders (50 females & 34 males; *M*age = 49.87 years, *SD*age = 14.86)) were asked to phonate a sustained /a:/ vowel at a comfortable pitch and loudness level for at least 5 s. The voice samples were simultaneously recorded in a soundproof booth through two devices that were placed at a 10 cm distance from the subjects’ mouths	Smartphone, external microphone	Healthy vs. pathological voice groups. A Samsung smartphone (Galaxy Note 3), and a high-quality external microphone (AKG Perception 220)	f0, jitter, shimmer, normalized noise energy (NNE), signal-to-noise ratio (SNR), and harmonics-to-noise ratio (HNR)	Dr. Speech	After splitting their sample and performing separate analyses for the healthy and pathological voices, they found in the healthy voice group significant differences for all the investigated acoustic voice parameters, except shimmer and f0, with the mean values from the external microphone recordings being higher. For the pathological voice group, no significant differences were found for the mean values of jitter, shimmer, and f0. For both groups, they showed significant correlations among the measured voice features reflecting pitch and amplitude perturbations (jitter and shimmer) and the features of voice signal turbulences (NNE, HNR, and SNR) captured both from the external and smartphone microphones
Lin et al., 2012.	11 healthy subjects (6 females & 5 males – *M*age = 41.8 years, *SD*age = 16.7) were simultaneously recorded in a quiet room through a smartphone and an external microphone. All subjects were asked to read six sentences. The smartphone was placed approximately at 13 cm distance from the subjects’ mouth, whereas the external microphone was approximately at 5 cm distance from the subjects’ mouth. Vowel segments were used to extract the investigated vocal features	Smartphone, external microphone	An iPhone smartphone (model A1303), and a high-quality external microphone (AKG C420)	f0, jitter, shimmer, signal-to-noise ratio (SNR), amplitude difference between the first two harmonics (H1 – H2), singing power ratio (SPR), and frequencies of formants one and two	TF32, Adobe Audition	The correlations between the vocal features captured by the smartphone and the external microphone were found to range from extremely to moderately high. The results showed a significant effect of the device used for shimmer, SNR, H1 – H2, and SPR and a significant device by vowel type interaction effect for shimmer and SPR in each vowel
Brown et al., 2020	Crowdsourced voice data were collected via the “COVID-19 Sounds App” (web-based, Android, iOS). Subjects were asked to cough three times, breathe deeply through their mouth three to five times, and read a short sentence appearing on the screen three times	Any device that connects to the internet (e.g., smartphone, laptop)	Tested positive vs. negative for COVID-19, reported symptoms	Duration, onset, tempo, period, root mean square energy, spectral centroid, roll-off frequency, zero-crossing, and mel-frequency cepstral coefficients (MFCCs) measures	Python (librosa), VGGish	Coughing sounds can distinguish COVID-19-positive from COVID-19-negative individuals with 80% precision. No device effects were tested or reported
Han et al., 2021	Crowdsourced voice data were collected via the “COVID-19 Sounds App” (web-based, Android, iOS). Subjects were asked to cough three times, breathe deeply through their mouth three to five times, and read a short sentence appearing on the screen three times	Any device that connects to the internet (e.g., smartphone, laptop)	Tested positive vs. negative for COVID-19, reported symptoms	Zero-crossing-rate (ZCR), root mean square frame energy, f0, harmonics-to-noise ratio (HNR), mel-frequency cepstral coefficients (MFCCs), prosodic, spectral, and voice quality features	openSMILE	When distinguishing positive tested individuals from negative ones without taking their symptoms into account, the model achieves a sensitivity and specificity of 62 and 74%, respectively. When distinguishing recently tested positive individuals from healthy controls without any symptoms, the ROC-AUC and PR-AUC both increase from around 75 to 79%. While the sensitivity and specificity are improved from 62 to 70%, and from 74 to 75%. No device effects were tested or reported
Parsa & Jamieson, 2001	Different microphones were tested on how they recorded three different acoustic stimuli: broadband, pure tone, and voice samples in a mini-anechoic chamber. Each of these signals was played back over a digital-to-analog converter The voice samples consisted of sustained samples of the vowel /a:/ from 53 healthy subjects (33 females & 20 males; age range 22–59 years) and 100 subjects with voice disorders (63 females & 37 males; age range 21–58 years). The exact distance between the microphone and the digital speaker is not reported	External microphones (specific devices were not reported)	One high-quality, expensive external microphone used for clinical purposes, and three cheaper external microphones used for clinical purposes (microphones’ brand and model are not reported). Healthy or pathological subjects	Four f0 perturbation measures, four amplitude perturbation measures, and four glottal noise measures	Not reported	Of the four f0 perturbation measures, the absolute jitter parameter was not significantly different for any of the microphone signals. All four of the amplitude perturbation measures were significantly different for all the microphones. The microphone affects classification accuracy between healthy and pathological voices. All amplitude perturbation measures were significantly different across microphones
Titze & Winholtz, 1993	4 subjects phonating a sustained /a:/ vowel and synthesized voice samples playing through a loudspeaker in a soundproof booth were used as the acoustic signal that was captured by different microphones at varying distances (4 cm, 30 cm, 1 m) and angles (0°, 45°, 90°). Each of the synthesized signals was 6 s and varied in terms of signal modulations (e.g., amplitude modulations, frequency modulations)	External microphones	Four professional-grade microphones (AKG 451EB CK22, AKG 451EB CK1, EV DO54, AKG D224E), and two consumer-grade microphones (Realistic 33-985, Realistic 33-1063). Different angles and distances	Amplitude and frequency measures	GLIMPES (Glottal Imaging by processing external signal)	Some consumer-grade microphones used in conjunction with the same equipment and analysis programs inflated the frequency perturbation to a range of 0.1–0.2% and amplitude perturbation to a range of 1–2%. When the microphone distance was changed from 4 cm to 1 m, perturbation measures significantly increased
Alsabek et al., 2020	Each subject was asked to cough four times, take a deep breath, and count from one to ten and instructed to have their head upright. The total collected number of samples used in this study was 42 [(7 COVID-negative speakers × 3 recordings) + (7 COVID-positive speakers × 3 recordings)]. The captured speech signal underwent pre-processing, which involved the removal of noise using PRAAT	Mobile phones (not further specified)	Tested positive vs. negative for COVID-19	Mel-frequency cepstral coefficients (MFCCs)	Not reported	The voice of subjects has shown a high correlation between COVID-negative and COVID-positive samples
This research	30 Subjects uttered 2 phrases in 3 emotional states while being recorded simultaneously via five common consumer-grade audio-recording devices. Participants were recorded using both low proximity to speaker microphones (i.e., smartphone, laptop, and studio microphone) placed away from the source (60 cm), and in high proximity to the speaker (i.e., lavalier and headset microphones) close to the source (15–20 cm)	Smartphone, laptop, studio microphone, headset, lavalier	Five distinct devices that were recording simultaneously: a studio microphone (Blue Yeti Logitech), a lavalier microphone (SmartLav+ Rode), a headset microphone (Beats by Dr. Dre EP), smartphone (Samsung A6), and a laptop (MacBook Pro 2017). To increase speaker variability, participants expressed three discrete emotions (neutral, happy, and sad) with two varying intonation types (phonetic amplification of “i” vs. “a”), and with or without wearing a headset	Mean f0, and mean amplitude	Python (Parselmouth)	Significant differences between recording devices (e.g., amplification of amplitude measure for high-proximity devices) which in turn led to lower predictive accuracy across an emotion prediction or biological sex prediction task

However, no research we are aware of has systematically examined whether using different audio-recording devices indeed produces reliable and valid voice data. The emerging methodological work focused predominantly on establishing correlational (as opposed to causal) evidence that different vocal features extracted on different audio-recording devices covary, i.e., increasing amplitude on one device corresponds to an increasing amplitude on another device (Guidi et al., 2015; Manfredi et al., 2017; Uloza et al., 2015).

While this initial correlational evidence is useful to establish that the directionality of the same measures assessed across different devices is consistent, it is also problematic for at least two important reasons. First, participants’ voice samples are typically analyzed to assess group differences and differences in magnitude (e.g., angry customers speak with a louder voice and greater variability of f0) (Li et al., 2017; Lin et al., 2012; Parsa & Jamieson, 2001). Thus, any differences driven by the device itself would bias group comparisons that rely on differences in magnitude between conditions or groups. Second, the dominant research design in prior methodological crowdsourced work is to compare participants without taking the recording device and the distance from the device into account (Brown et al., 2020; Han et al., 2021; Shimon et al., 2021), which makes it impossible to differentiate between device, distance, and speaker-related effects. Also recently published, large-scale datasets to predict the presence of a COVID-19 infection did not report or control for differences in recording device or recording setting, even though the prediction accuracy varied greatly both within and between these crowd-sourced datasets, highlighting that unexplored factors may help to boost accuracy (i.e., “Coswada” – Sharma et al., 2020; “Covid-19 Sounds App” – Xia et al., 2021; “COVID-19 Voice Detector” & “Vocalis-Health App” – Shimon et al., 2021).

In sum, to the best of our knowledge, no research has carefully and systematically examined whether using different audio-recording devices indeed produces valid and reliable audio data. The objective of this research is to provide a first systematic analysis of the same person’s speech signals, recorded on a variety of different consumer-grade audio-capturing devices. We demonstrate how the characteristics of the recording device can drastically impact the extracted vocal features and substantive conclusions drawn from this data.

### Environmental conditions and microphone placement

To capture accurate and consistent audio signals, the recording conditions play a pivotal role (Krik et al., 2019). Factors such as the environment (e.g., background noise) (Parsa & Jamieson, 2001) and placement (e.g., distance) of the audio-recording device (Titze & Winholtz, 1993) directly affect the captured audio signal. A variation of these factors can negatively impact the accuracy of vocal measurements and lead to inaccurate conclusions.

The recording environment can significantly vary among participants in crowdsourced online experiments. Reflections from sounds hitting the walls, as well as background noise, can interfere with the targeted acoustic signal, altering, in turn, the accuracy of the extracted vocal measurements (Titze, 1995).

Another crucial factor affecting the acoustic signal is the placement of the microphone relative to the sound source. Specifically, increasing the distance between the microphone and the sound source can lead to a weaker signal and less accurate f0 and amplitude perturbation measures (Švec & Granqvist, 2010). Furthermore, when recording with microphones not capable of capturing very high sound levels (up to 147 dB), a short distance combined with a loud source can cause signal distortion (Švec & Granqvist, 2010). Besides distance, the angle between the microphone and the source further influences the captured acoustic signal. Specifically, f0 and amplitude perturbation measures show less accuracy with increasing angles (Parsa & Jamieson, 2001). Distance and angle can also produce unwanted interaction effects. A microphone located in high proximity at the side of the mouth can systematically distort spectral and sound pressure level measurements (Titze & Winholtz, 1993).

### How audio-recording devices shape acoustic signal measurements

The microphone characteristics of audio-recording devices can also affect the captured acoustic signal (Kisenwether & Sataloff, 2015), altering vocal measurements such as the frequency spectrum (Parsa & Jamieson, 2001) and amplitude perturbation measures (Titze & Winholtz, 1993).

Prior research has attempted to evaluate the recording performance of different microphones and recommends the usage of a high-quality microphone with high impedance, a flat frequency response, and a broad frequency range (20–20,000 Hz) (Krik et al., 2019). Nevertheless, participants in crowdsourced online experiments do not have the luxury of owning a piece of high-end recording equipment, and thus the majority of study participants use consumer-grade audio-recording devices during experiments. These inaccuracies are prominent in the captured f0 and amplitude perturbation measures, which were estimated to be approximately three times higher when recording with consumer-grade microphones compared to professional ones (Titze & Winholtz, 1993). Differences in the captured acoustic signal have also been found between different types and polar patterns of audio-recording devices. A studio-quality condenser microphone yields significantly better results compared to a dynamic microphone, with the latter resulting in higher captured f0 and amplitude perturbations (Parsa & Jamieson, 2001). Cardioid condenser microphones are more accurate and had the least effects on f0 and amplitude perturbation measures of voice recordings due to the microphone’s greater sensitivity (Titze & Winholtz, 1993).

These differences between audio-recording devices can have important practical implications. For instance, in a clinical context, variation in microphones’ characteristics can negatively impact diagnosing pathological from normal voices (Parsa & Jamieson, 2001), highlighting the importance of high consistency across recordings.

In sum, the objective of this research is to systematically compare voice recordings collected via common audio-recording devices and provide causal evidence to which extent conclusions from such audio data are valid across devices and different voice expression scenarios (e.g., different types of discrete emotions and intonation patterns).

### Fundamental components of acoustic speech signals

The human speech is a complex acoustic signal that can be decomposed and quantified into four fundamental domains: time, amplitude, fundamental frequency, and spectrum (Hildebrand et al., 2020). Examples of primary vocal features in these domains include, for example, duration and speech rate in the *time domain*, average intensity (i.e., loudness) and standard deviation of the intensity (loudness variability) in the *amplitude domain*, average f0 (i.e., pitch) and standard deviation of f0 (i.e., pitch variability) in the *fundamental frequency domain*, and jitter and shimmer (i.e., perturbation measures) in the *spectral domain* (Hildebrand et al., 2020; Juslin & Laukka, 2003). The feature extraction across these four dimensions and the analysis of each specific vocal feature of a speaker reveal nuanced insight such as moment-to-moment variation of the speaker’s emotional state (Johnstone & Scherer, 2000; Scherer et al., 1991). For instance, prior research has shown that enhanced levels of enjoyment are associated with increased f0 (Scherer et al., 1991), whereas sadness is linked to lower average intensity (Abelin & Allwood, 2000) indicated by a noticeably reduced loudness of a sad individual (for a more extensive review of the literature, see Hildebrand et al., 2020; Juslin & Laukka, 2003).

The two most defining dimensions to assess acoustic markers in the human voice are the speaker’s fundamental frequency and amplitude because features in the spectral domain are predominantly examined in pathological speech settings and time is often taken as a control measure (Erdogdu Sakar et al., 2017; Pellowski, 2010; Sharma et al., 2020; Uloza et al., 2015; van Nuffelen et al., 2009; Wang et al., 2016). We, therefore, focus on differences in speakers’ amplitude and fundamental frequency throughout this paper, assessing how capturing each of these two key vocal features (e.g., amplitude and fundamental frequency) varies as a function of the recording device.

## Design and procedures

### Data collection

We collected a total of 1,800 voice samples using a nested within-subjects experimental design. A total of 30 participants were recruited (*M*_Age_ = 24.73, *SD*_Age_ = 5.09, 50% females) at the behavioral lab of a major European university and assigned to five distinct recording device conditions (baseline studio-quality microphone, lavalier microphone, headset microphone, smartphone, laptop). The recording device condition was of primal interest in the current research as the core question is whether the validity of the extracted features varies as a function of the recording device. The five recording devices were specifically selected as the most common and representative devices used in prior research (see Table 1).

To create a realistic recording setting with sufficient speaker variation, the recording device condition was crossed with three additional factors: *Intonation type* (phonetic amplification of “i” [as in “beer”] vs. “a” [as in “bar”]), wearing a *headset* (vs. not), and a *discrete emotion condition* (same phrase issued either as neutral, happy, or sad). These conditions were assessed to capture a broad range of an individual’s true range of auditory speech signals when assessing the impact of our key factor of interest (recording device).

We altered the *intonation type* to create recording settings that allow us to capture the full spectrum of the person’s natural speaking range without artificially capturing a disproportionate number of vowels that are either associated with higher frequencies (“i”) compared to vowels with lower frequencies (“u”, “a”) (Coleman, 1971; Maurer, 2016). The presence or absence of *headsets* was introduced to increase the realism of our design, given that over 50% of people use headsets daily or several times a week, and is common in laboratory recording settings (Statista, 2017). Prior work has shown that wearing hearing protection of any kind can cause attenuation and occlusion effects, which can elicit inadvertent vocal adjustments by the speaker (Giguère et al., 2017). The three *discrete emotion conditions* finally served the purpose to enhance intraspeaker variability and to assess the prediction accuracy in tasks in which we know the ground truth (i.e., participants will be explicitly instructed to speak in a happy, neutral, or sad voice). We report more expansive results in the Web Appendix (e.g., presence vs. absence of headsets) and focus on our key factor of interest in what follows (i.e., recording device type).

Across all sessions, participants were comfortably seated on a chair in front of a desk. The lavalier microphone was attached approximately at chest level, pointing toward their mouth (visual instructions provided). All other devices were positioned at an equal distance from the participant. In the headset present condition, participants were wearing headphones throughout the task while in the headset absent condition, the headphones were placed around the participants’ neck. The intonation type manipulation was inspired by prior work in linguistics (Hellbernd & Sammler, 2016), to assess a speaker’s phonetic profile range by saying two short phrases: “*I go to the bar*” and “*I drink a beer*”. Participants were instructed to say each of the phrases in a neutral, happy, or sad voice. To avoid ordering effects, participants started with the neutral emotion, followed by a random order of the happy or sad emotion condition. Finally, we provided written instructions in addition to the verbal instructions by the experimenter at the beginning of the session. Once the participant understood the task, the experimenter started the recording across all devices and left the room. The data collection was conducted individually and lasted between 10 and 15 min per subject. At the end of the experiment, every participant was debriefed and received a compensation of 10 Swiss Francs.

### Measurements

As highlighted earlier, we assessed two fundamental features of the human voice (see Hildebrand et al., 2020; Juslin & Laukka, 2003 for a review): The fundamental frequency (f0) and the amplitude of the speaker. As we briefly summarize in what follows, these two defining characteristics of the human voice vary systematically both between individuals (such as higher f0 among females versus males) and also across contexts (such as typically greater amplitude of happy versus sad vocal expressions).

#### f0

The f0 is defined as the lowest frequency of a periodic waveform. Its perceptual correlate is the pitch of a person with low (high) values of f0 making a voice sound deeper (shriller) (Oxenham, 2012). Prior emotion research has found that specific emotional states are associated with corresponding f0 values; for instance, anger and happiness have been linked to higher f0, whereas fear seems to be linked to lower f0. Moreover, f0 is considered a key factor in predicting the speaker’s sex (Henton, 1995) with f0 values of an adult female voice ranging between 165 and 255 Hz and f0 values of an adult male ranging between 85 and 155 Hz (Watson, 2019).

#### Amplitude

Amplitude is defined as the displacement of a soundwave from its equilibrium position (Everest & Pohlmann, 2015); higher (lower) amplitude voices are perceived as louder (quieter). Similarly to f0, different amplitude levels in the human voice have been found to correlate with discrete emotional states. For example, anger is often associated with higher amplitude levels such as when a person is shouting (Clark, 2005), and sadness with lowered amplitude levels (Hildebrand et al., 2020; Juslin & Laukka, 2003).

### Materials and recording setup

As explained in the Data collection section, participants were simultaneously recorded by five different consumer-grade audio-recording devices that are commonly used in current crowdsourced research studies. Specifically, we used the built-in microphone of an Android smartphone (Samsung A6), the built-in microphone of an Apple laptop (MacBook Pro, 2017), a lavalier microphone connected to a smartphone, the microphone of a headset (Beats by Dr Dre EP) connected to a smartphone, and a non-portable studio-quality microphone (Blue Yeti) connected to a desktop computer (serving as a baseline condition). To simulate real-life conditions, the lavalier and headset microphones were placed in normal usage conditions (approximately at chest level, with a 15–20 cm distance to the mouth of a participant), whereas the other four microphones were placed in front of the speaker with each microphone facing toward the participant (approximately 60 cm). Audio recordings for the smartphone, lavalier, and headset conditions, were captured via the “Easy Voice Recorder Pro” app, and the studio-quality microphone and laptop conditions were recorded using Audacity. All voice files were recorded with the same sampling rate of 44,100 Hz and exported in WAV 32-bit float PCM format (see Table 2 for a summary). Web Appendix C provides an overview of the technical specifications of each microphone used in the current research (Švec & Granqvist, 2010).

**Table 2:** Hardware setup & recording details

Audio-recording device	Brand	Model	Proximity to participants	Software tool to record	Peripheral / built-in	Audio file type	Sampling rate
Lavalier	Rode	SmartLav+	High	Easy voice recorder pro app	Peripheral	WAV	44,100 Hz
Headset	Apple	Beats by Dr. Dre EP	High	Easy voice recorder pro app	Peripheral	WAV	44,100 Hz
Studio (Baseline)	Logitech	Blue Yeti	Low	Audacity	Peripheral	WAV	44,100 Hz
Smartphone	Samsung	A6	Low	Easy voice recorder pro app	Built-in	WAV	44,100 Hz
Laptop	Apple	MacBook Pro, 2017	Low	Audacity	Built-in	WAV	44,100 Hz

### Data processing and analysis

The extraction and estimation of the vocal measurements (mean f0 & mean amplitude) were performed using Parselmouth, a PRAAT implementation using Python. We used one-way repeated-measures ANOVAs to test for differences in f0 and amplitude across audio-recording devices. To probe significant effects, we used post-hoc contrasts with Tukey HSD adjustment to test condition differences. Furthermore, we used two-way repeated-measures ANOVAs to test interaction effects between audio-recording devices and the speaker’s biological sex or audio-recording devices and simulated emotions. In the presence of a significant interaction effect, we assessed subsequent condition differences with the same error adjustment as mentioned earlier (i.e., Tukey HSD). As the key focus of this research is on recording device effects, we report tangential analyses in the Web Appendix (e.g., differences when wearing a headset vs. not). We share all code, data processing steps, and analyses using an OSF repository (https://osf.io/9g87v/?view_only=b26a8ee893f04d43a12ac965250e2438).

### Reference microphone

To evaluate how accurately common audio-recording devices record amplitude and f0, we used a studio-quality microphone as a baseline. Our baseline microphone supports a wide frequency range from 20 Hz up to 20,000 Hz with an almost flat frequency response within the human speaking frequency range. The microphone has a dynamic range of 114 dB with a maximum sound pressure level rating of 120 dB (loudness comparable with the noise levels of a chainsaw). We used the cardioid directionality pattern to pick up the audio signal (front direction) to reduce ambient noise and the reverberation sound in the room (Švec & Granqvist, 2010), since cardioid microphones provide a higher signal quality (Vogel & Morgan, 2009) and are considered the best choice to record voices for clinical purposes (Baken & Orlikoff, 2000).

## Results

### Amplitude and f0 across audio-recording devices

#### Correlations across recording devices

**Fig. 1:**
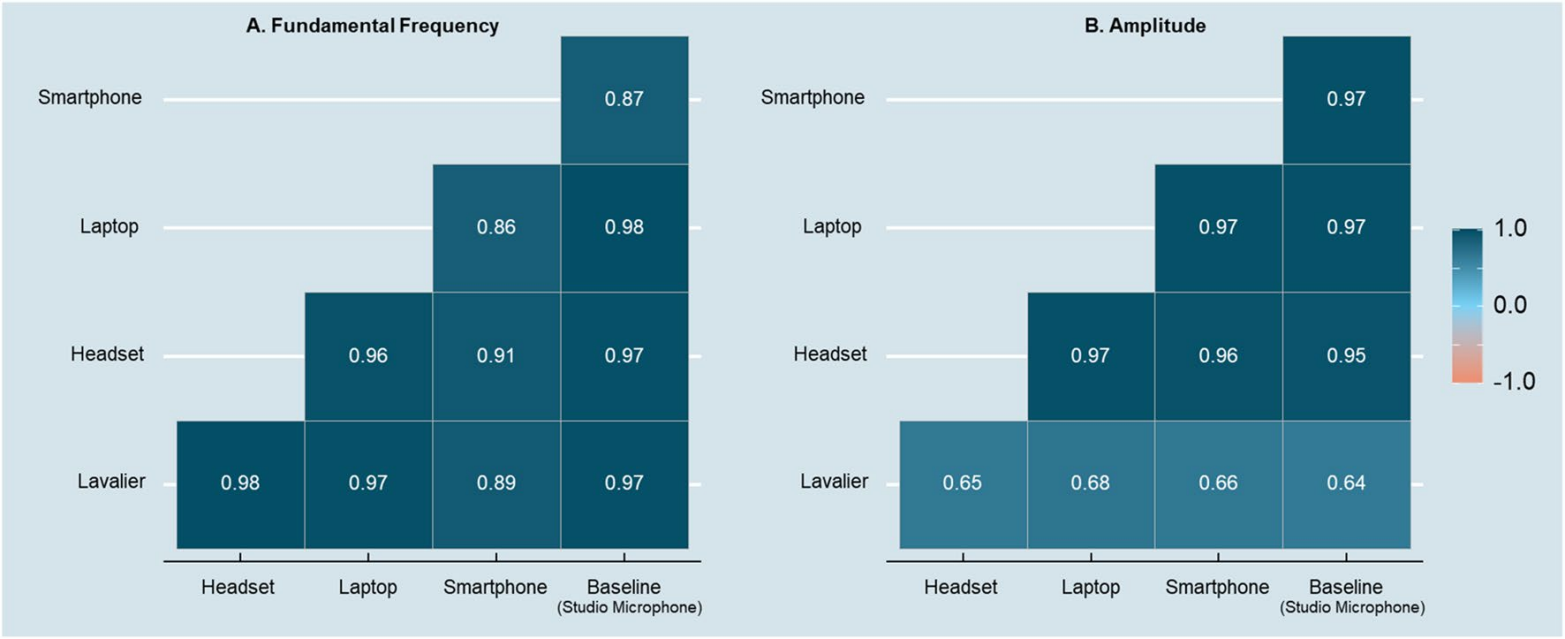
Correlograms of f0 (**A**) and amplitude (**B**) among audio-recording devices

Before assessing group-level differences across recording devices, we first assess collinearity across our key vocal measures across recording devices. As illustrated in the correlogram of Fig. 1A, we find that all f0 measures were significantly correlated (average correlation coefficient: *r* = .94, *p* < .001; 95% CI = [.92; .95]). The size and magnitude of the correlation are in line with prior work (e.g., Guidi et al., 2015). We observed similar effects for amplitude, and as shown in the correlogram of Fig. 1B, the amplitude measures across devices were significantly, and positively correlated (*r* = .84, *p* < .001; 95% CI = [.81; .87]). A Farrer–Glauber test to assess the covariation of the entire set of variables further confirmed the collinearity of both the f0 (Farrar chi-square = 2,808.27) as well as the amplitude features (Farrar chi-square = 3,155.92).

In summary, the size and direction of correlations between vocal features are in a similar range compared to prior work. However, as we outline in what follows, even though the same vocal features may covary across recording devices, any differences driven by the device itself may bias group comparisons that rely on differences in magnitude between groups.

#### Amplitude

**Fig. 2:**
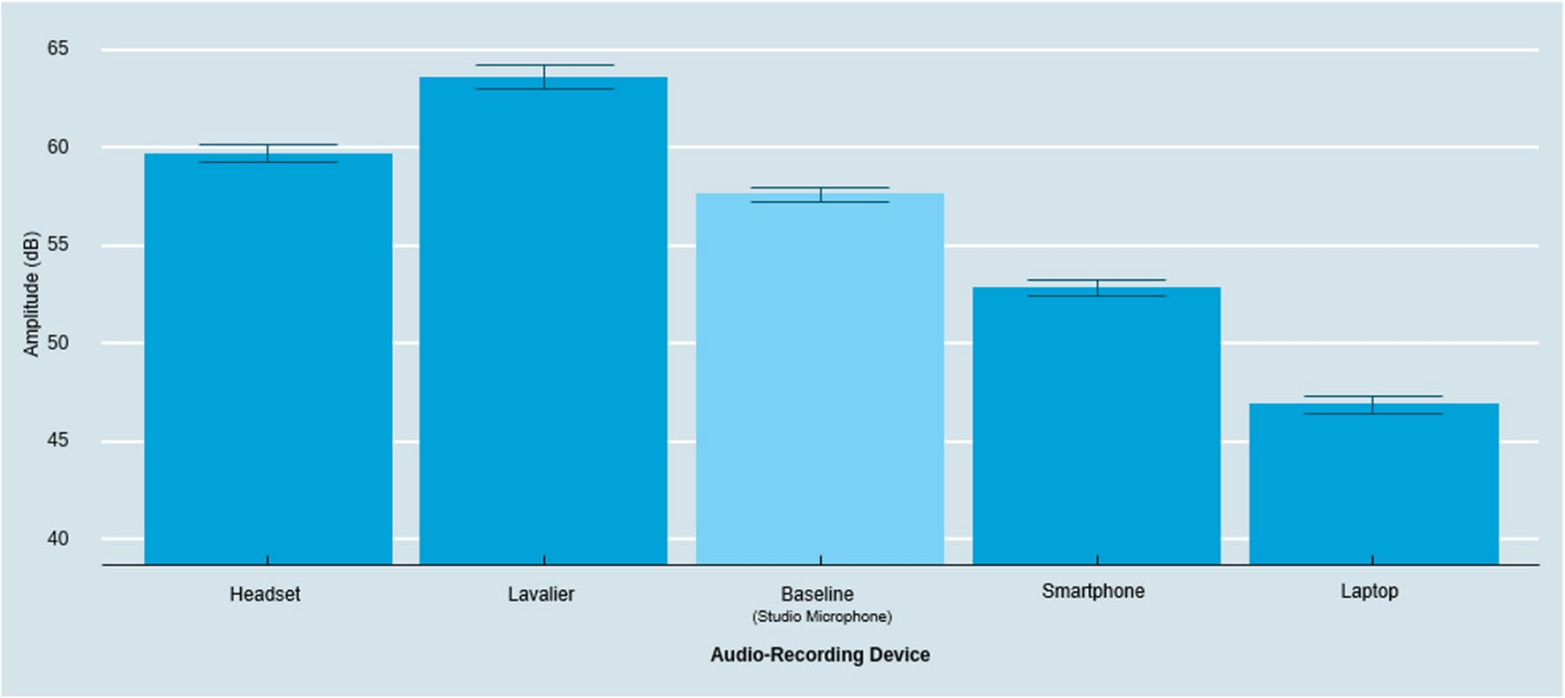
Amplitude across audio-recording devices. *Note*: *Error bars* represent a 95% confidence interval around the mean

We observed a significant effect of the audio-recording device on the amplitude of the speakers’ voice (*F*(4, 116) = 148.70, *p* < .001, η_p_^2^ = .84). As shown in Fig. 2, follow-up contrasts with Tukey HSD correction for multiple comparisons confirmed that all devices captured significantly different levels of amplitude (marginally significant when comparing baseline versus headset: *M*_Headset_ = 59.73 dB, *M*_Baseline_ = 57.67 dB, *t* = 2.73, *p* = .06). The lavalier microphone captured significantly higher amplitude compared to all low-proximity devices (*M*_Lavalier_ = 63.68 dB, *M*_Smartphone_ = 52.90 dB, *M*_Laptop_ = 46.91 dB, Lavalier – Smartphone: *t* = 10.77, *p* < .001; Lavalier – Laptop: *t* = 16.76, *p* < .001; Lavalier – Baseline: *t* = 5.99, *p* < .001), as well as compared to the other high-proximity recording devices (Lavalier – Headset:* t* = 3.94, *p* < .001). Comparing solely between the low-proximity devices, both the smartphone and laptop captured significantly lower amplitude compared to the baseline (Laptop – Baseline: *t* = – 14.34, *p* < .001; Smartphone – Baseline: *t* = – 6.37, *p* < .001). Finally, the laptop captured significantly lower amplitude than all other devices (Laptop – Smartphone: *t* = – 7.98, *p* < .001, Laptop – Lavalier: *t* = – 22.32, *p* < .001, Laptop – Baseline: *t* = – 14.34, *p* < .001, Laptop – Headset: *t* = – 17.08, *p* < .001).

**Fig. 3:**
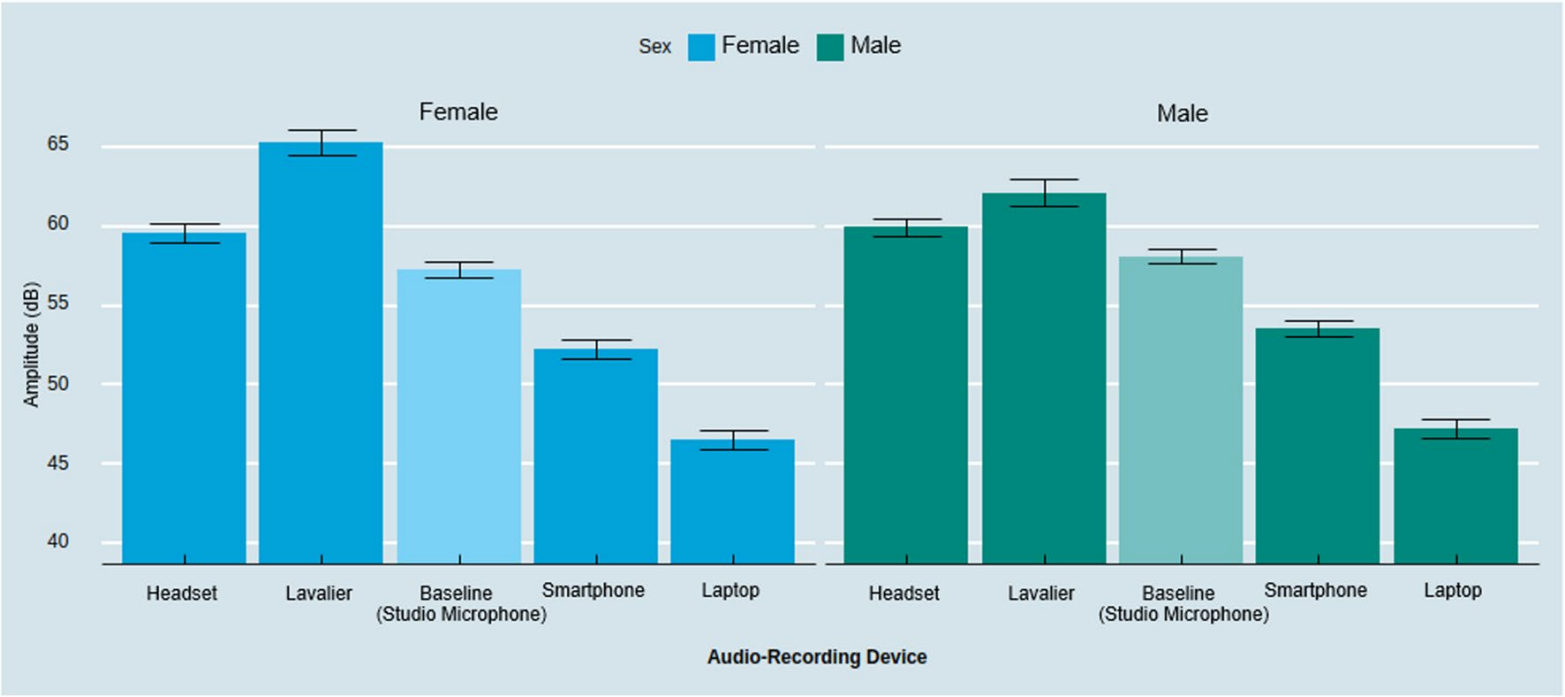
Amplitude across audio-recording devices by biological sex. *Note*: *Error bars* represent a 95% confidence interval around the mean

Next, we assessed whether these systematic differences vary between male and female participants. Overall, the absolute differences between recording devices remained robust and in the same direction as reported in the preceding section (see Figs. 2 and 3). As an exploratory side result, we found a significant interaction effect between the audio-recording device and the speaker’s biological sex (*F*(4, 112) = 3.14, *p* < .05, η_p_^2^ = .10). Probing the interaction revealed that, for males, the lavalier microphone did not capture a significantly different amplitude than the headset (*M*_Lavalier & Male_ = 61.71 dB, *M*_Headset & Male_ = 59.55 dB, *t* = 2.11, *p* = .52), while it did for females (*M*_Lavalier & Female_ = 65.62 dB, *M*_Headset & Female_ = 59.91 dB, *t* = 5.57, *p* < .001), indicating that differences in amplitude in high-proximity audio-recording devices are pronounced for female participants (see Fig. 3). This is consistent with a non-constant frequency response which can lead to enhanced sensitivity of higher frequencies and eventually higher gain at these frequencies (Beacham, 2018; Saeedivahdat et al., 2010).

#### f0

**Fig. 4:**
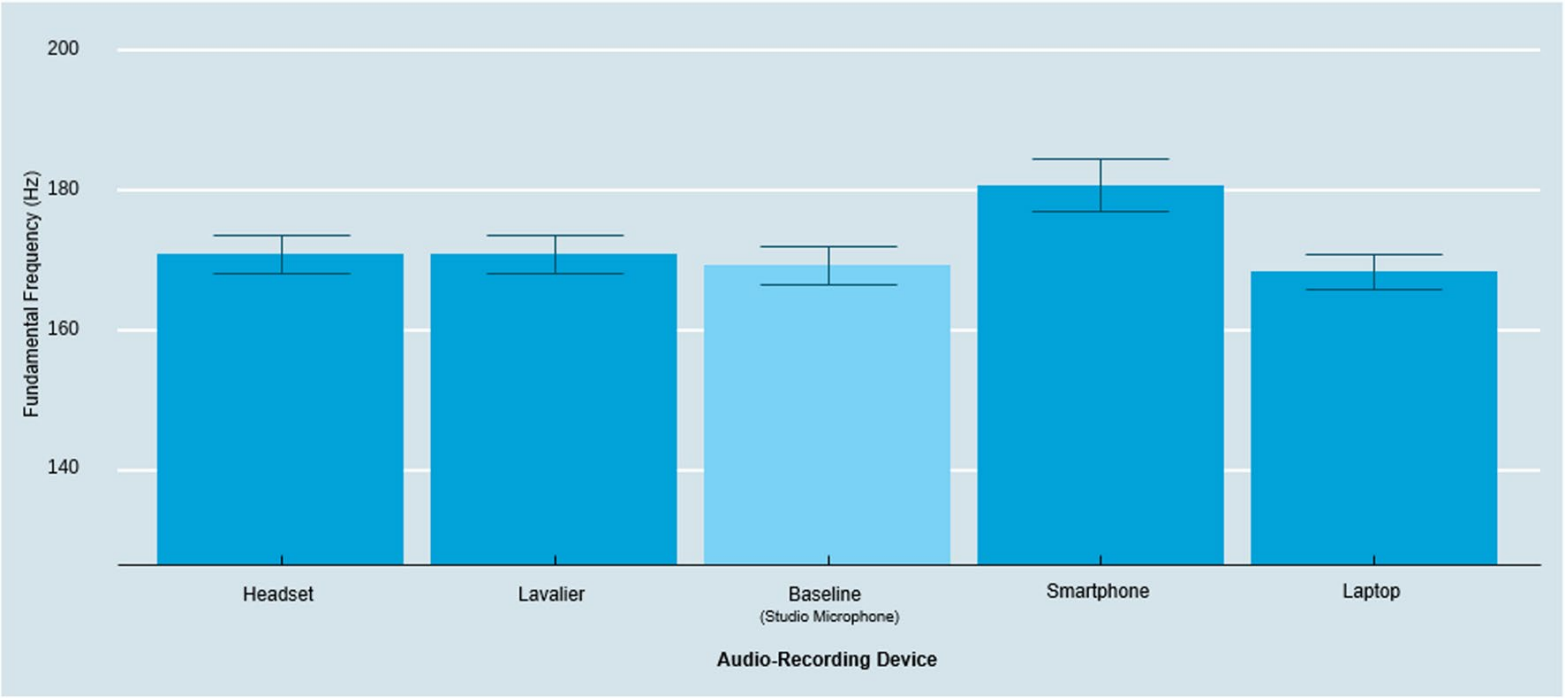
Fundamental frequency across audio-recording devices. *Note*: *Error bars* represent a 95% confidence interval around the mean

The type of audio-recording device also has a significant effect on capturing speakers’ f0 (*F*(4, 116) = 9.82, *p* < .001, η_p_^2^ = .25). As illustrated in Fig. 4, follow-up contrasts with Tukey HSD correction revealed that the smartphone captured a significantly higher f0 than the baseline and compared to all other audio-recording devices (*M*_Smartphone_ = 180.70 Hz, *M*_Laptop_ = 168.42 Hz, *M*_Baseline_ = 169.31 Hz, *M*_Headset_ = 170.88 Hz, *M*_Lavalier_ = 170.86 Hz; Smartphone – Baseline:* t* = 5.09, *p* < .001; Smartphone – Laptop: *t* = 5.49, *p* < .001; Smartphone – Lavalier: *t* = 4.40, *p* < .001; Smartphone – Headset: *t* = 4.39, *p* < .001). We found no significant differences between the other conditions (Lavalier – Headset: *t* = – .02, *p* = 1.00; Lavalier – Laptop: *t* = 2.44, *p* = .81; Lavalier – Baseline: *t* = .69, *p* = .96; Headset – Laptop: *t* = 1.10, *p* = .81; Headset – Baseline: *t* = .70, *p* = .96; Laptop – Baseline: *t* = – .89, *p* = .99).

**Fig. 5:**
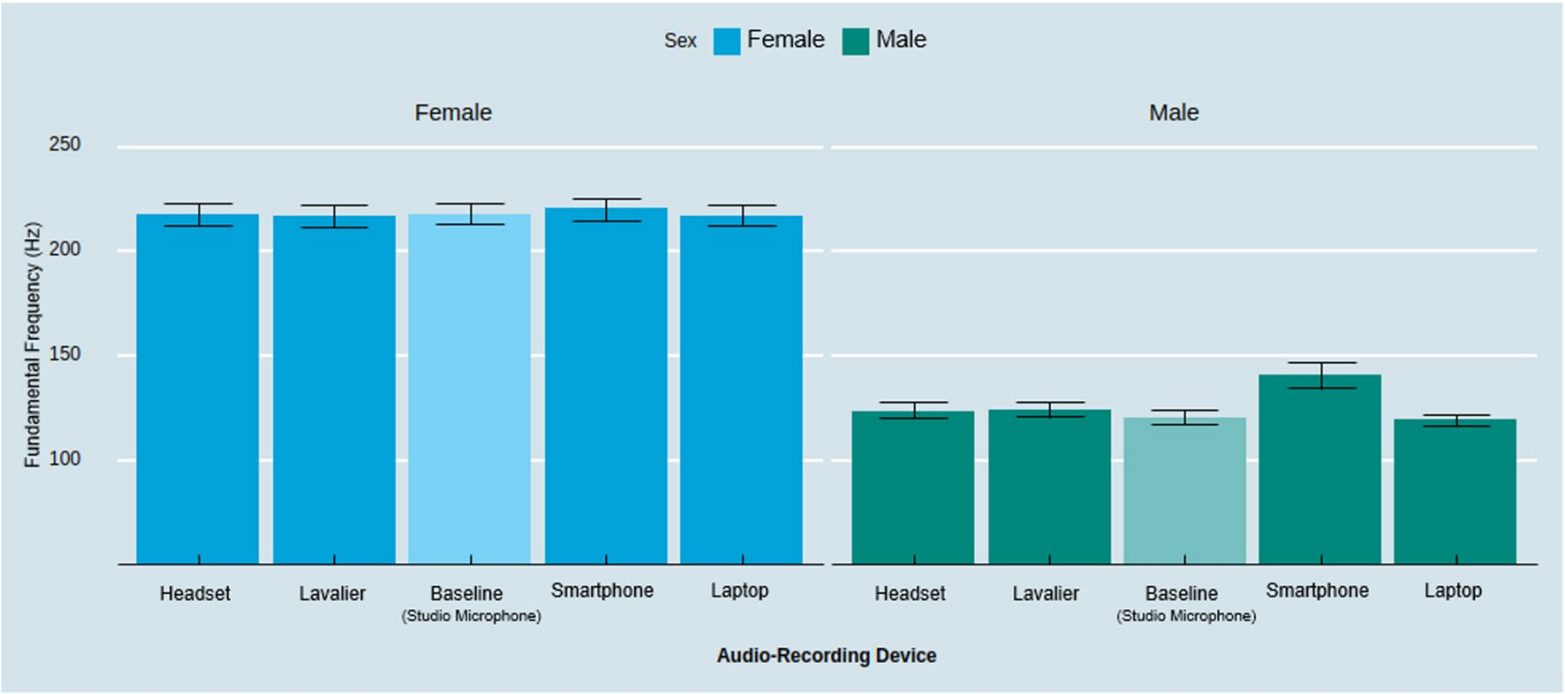
Fundamental frequency across audio-capturing devices by biological sex. *Note*: *Error bars* represent a 95% confidence interval around the mean

Further conditioning the effect based on the speaker’s biological sex, we observed a significant interaction effect between audio-recording devices and sex (*F*(4, 112) = 6.92, *p* < .001, η_p_^2^ = .20). Follow-up contrasts with Tukey HSD correction revealed no significant differences on f0 between devices for females; but it did for males (see Fig. 5). The smartphone captured significantly higher f0 for males compared to all other devices (*M*_Smartphone & Male_ = 141.06 Hz, *M*_Laptop & Male_ = 119.42 Hz, *M*_Baseline & Male_ = 120.64 Hz, *M*_Headset & Male_ = 123.96 Hz, *M*_Lavalier & Male_ = 124.60 Hz, Smartphone_Male_ – Baseline_Male_:* t* = 20.42, *p* < .001, Laptop_Male_ – Smartphone_Male_:* t* = 21.64, *p* < .001, Smartphone_Male_ – Lavalier_Male_: *t* = 16.47, *p* < .001, Smartphone_Male_ – Headset_Male_: *t* = 17.11, *p* < .001). Simply put, recording with the smartphone device led to amplified measurements of the f0 for male participants. Although this finding is exploratory, this variation might be driven by known differences such that the built-in microphones of common smartphone manufacturers rely on so-called “high-pass filters” which only allow frequencies above a certain level to “pass” through as an audio signal with the purpose to reduce proximity effects when a person is close to the recording device or microphone (Clifford & Reiss, 2011; McAllister, 2022).

### Emotion expression task

The observed systematic differences in amplitude and f0 between the audio-recording devices raise the question of whether these devices can capture differences in amplitude and f0 between the three discrete emotion expression conditions (neutral, happy, sad emotion expression).

#### Amplitude

**Fig. 6:**
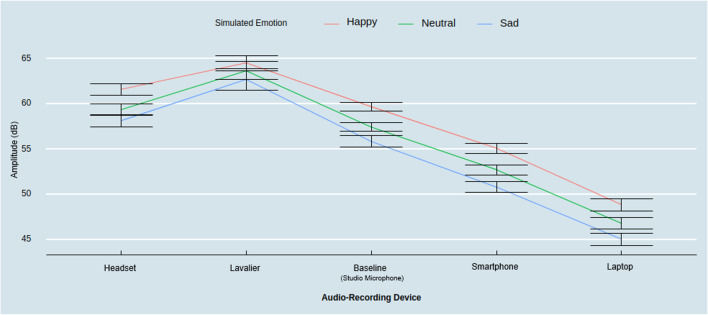
Amplitude across audio-recording devices by emotional state. *Note*: *Error bars* represent a 95% confidence interval around the mean

First, we found a significant interaction between audio-recording devices and emotion condition on amplitude (*F*(8, 232) = 16.00, *p* < .001, η_*p*_^*2*^ = .36). Follow-up contrasts with Tukey HSD correction revealed that all devices captured significantly higher amplitude between happy and sad states (*M*_Laptop & Happy_ = 48.88 dB, *M*_Baseline & Happy_ = 59.70 dB, *M*_Smartphone & Happy_ = 55.12 dB, *M*_Lavalier & Happy_ = 64.54 dB, *M*_Headset & Happy_ = 61.62, *M*_Laptop & Sad_ = 45.05 dB, *M*_Baseline & Sad_ = 55.86 dB, *M*_Smartphone & Sad_ = 50.82 dB, *M*_Lavalier & Sad_ = 62.73 dB, *M*_Headset & Sad_ = 58.16 dB; Laptop_Happy_ – Laptop_Sad_: *t* = 7.95, *p* < .001; Baseline_Happy_ – Baseline_Sad_: *t* = 7.97, *p* < .001; Smartphone_Happy_ – Smartphone_Sad_: *t* = 8.93, *p* < .001; Lavalier_Happy_ – Lavalier_Sad_: *t* = 3.74, *p* < .05; Headset_Happy_ – Headset_Sad_: *t* = 7.16, *p* < .001). Similarly, all the devices, except the lavalier (*M*_Lavalier & Neutral_ = 63.72 dB, *t* = 1.71, *p* = .93), captured significantly higher amplitude between happy and neutral states (*M*_Laptop & Neutral_ = 46.79 dB, *M*_Baseline & Neutral_ = 57.47 dB, *M*_Smartphone & Neutral_ = 52.75 dB, *M*_Headset & Neutral_ = 59.41 dB; Laptop_Happy_ – Laptop_Neutral_: *t* = 4.33, *p* < .01; Baseline_Happy_ – Baseline_Neutral_: *t* = 4.63, *p* < .01; Smartphone_Happy_ – Smartphone_Neutral_: *t* = 4.93, *p* < .001; Headset_Happy_ – Headset_Neutral_: *t* = 4.59, *p* < .01). Only the laptop and smartphone captured significantly higher amplitude between the neutral and the sad state (Laptop_Sad_ – Laptop_Neutral_: *t =* 3.62, *p* < .05; Smartphone_Sad_ – Smartphone_Neutral_: *t* = 4.00, *p* < .05), while the baseline device captured a marginally significant higher amplitude in the neutral compared to the sad state (Baseline_Sad_ – Baseline_Neutral_: *t* = 3.34, *p = *.08) (see Fig. 6).

These findings support the idea that when the differences in amplitude are more extreme (e.g., between happy and sad states), they can be captured by any device, whereas, in the context of less extreme amplitude differences (e.g., between happy and neutral or sad and neutral states), the high-proximity microphones are unable to reliably detect them due to the greater amplitude as reported previously. Thus, the stronger the amplification of the amplitude dimension (as in high-proximity recording devices), the smaller the differences that can be reliably captured between discrete emotion conditions. Finally, we found no significant three-way interaction among recording devices, biological sex, and simulated emotion on amplitude (*F*(8, 224) = 1.15, *p* = .33, η_*p*_^*2*^ = .04).

#### f0

Next, zooming in on f0 as a function of the audio-recording device and emotion condition, we found a significant interaction effect (*F*(8, 232) = 2.29, *p* < .05, η_p_^2^ = .07). Follow-up contrasts with Tukey HSD correction revealed that the smartphone captured significantly higher f0 compared to all other devices in the happy (*M*_Baseline & Happy_ = 188.38 Hz, *M*_Smartphone & Happy_ = 203.35 Hz, M_Laptop & Happy_ = 186.72 Hz, M_Headset & Happy_ = 190.56 Hz, M_Lavalier & Happy_ = 190.96 Hz; ; Smartphone_Happy_ – Baseline_Happy_: *t* = 5.76, *p* < .001; Smartphone_Happy_ – Laptop_Happy_: *t* = 6.40, *p* < .001; Smartphone_Happy_ – Headset_Happy_: *t* = 12.79, *p* < .001; Smartphone_Happy_ – Lavalier_Happy_: *t* = 12.38, *p* < .001) and the neutral state (*M*_Baseline & Neutral_ = 163.74 Hz, *M*_Smartphone & Neutral_ = 175.50 Hz M_Laptop & Neutral_ = 162.99 Hz, M_Headset & Neutral_ = 165.35 Hz, M_Lavalier & Neutral_ = 165.93 Hz; Smartphone_Neutral_ – Baseline_Neutral_: *t* = 4.52, *p* < .001; Smartphone_Neutral_ – Laptop_Neutral_: *t* = 12.51, *p* < .001; Smartphone_Neutral_ – Headset_Neutral_: *t* = 10.15, *p* < .001; Smartphone_Neutral_ – Lavalier_Neutral_: *t* = 9.57, *p* < .001), but not for the sad state (*M*_Baseline & Sad_ = 155.82 Hz, *M*_Smartphone & Sad_ = 163.26 Hz, M_Laptop & Sad_ = 155.55 Hz, M_Headset & Sad_ = 156.73 Hz, M_Lavalier & Sad_ = 155.69 Hz; Smartphone_Sad_ – Baseline_Sad_: *t* = 2.86, *p =* .22; Smartphone_Sad_ – Laptop_Sad_ *t* = 2.97, *p* = .18; Smartphone_Sad_ – Headset_Sad_: *t* = 2.51, *p =* .18; Smartphone_Sad_ – Lavalier_Sad_: *t* = 2.92, *p =* .20).

**Fig. 7:**
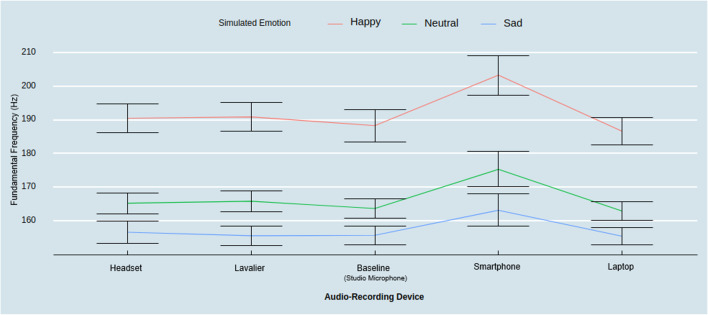
Fundamental frequency across audio-recording devices by emotional state. *Note*: *Error bars* represent a 95% confidence interval around the mean

Comparisons among the three simulated emotions using a within-device level analysis revealed that all devices captured higher f0 contrasting the happy and sad state (Baseline_Happy_ – Baseline_Sad_: *t* = 9.18, *p* < .001; Laptop_Happy_ – Laptop_Sad_: *t* = 8.79, *p* < .001; Smartphone_Happy_ – Smartphone_Sad_: *t* = 11.30, *p* < .001; Lavalier_Happy_ – Lavalier_Sad_: *t* = 9.95, *p* < .001; Headset_Happy_ – Headset_Sad_: *t* = 9.54, *p* < .001), as well as when contrasting the happy and neutral states (Baseline_Happy_ – Baseline_Neutral_: *t* = 9.18, *p* < .001; Laptop_Happy_ – Laptop_Neutral_: *t* = 6.69, *p* < .001; Smartphone_Happy_ – Smartphone_Neutral_: *t* = 7.85, *p* < .001; Lavalier_Happy_ – Lavalier_Neutral_: *t* = 7.06, *p* < .001; Headset_Happy_ – Headset_Neutral_: *t* = 7.11, *p* < .001). No recording device captured significantly different f0 values when contrasting the sad and neutral states, however, the smartphone reached marginally significant levels (Smartphone_Sad_ – Smartphone_Neutral_: *t* = 3.45, *p* = .06) (see Fig. 7). Finally, we found no significant three-way interaction among devices, biological sex, and emotion on f0 (*F*(8, 224) = 1.23, *p* = .28, η_*p*_^*2*^ = .04). In summary, discrete emotion differences at the within-device level can be reliably captured assessing the f0 of the speaker, except for the significantly amplified f0 in the smartphone recording condition.

### Emotion classification

Next, we tested how well we can predict participants’ expressed emotion condition from the vocal measurements across all audio-recording devices. Given the systematic differences in amplitude and f0 as a function of the recording device, the recording device may systematically impact both the average prediction accuracy of the expressed emotion from the speakers’ vocal measurements as well as our substantive conclusions. To assess the backtesting validity of the recording devices, we trained a random forest to predict the speaker’s emotion condition using amplitude and f0, for each of the different audio-recording devices while controlling for participants’ biological sex, amplitude perturbations, and f0 perturbations (Farrús et al., 2007). We used five-fold subject-wise cross-validation to assess the predictive accuracy of the different models.

**Fig. 8:**
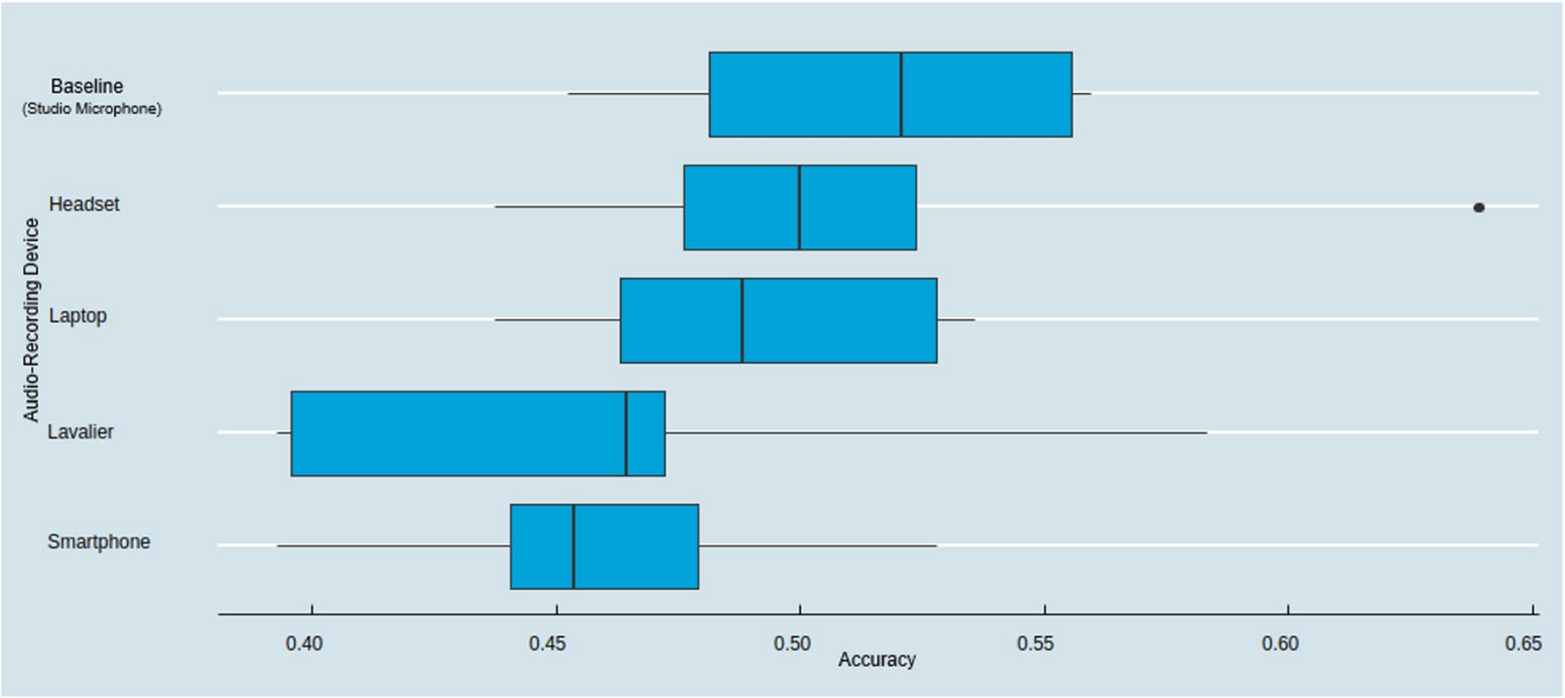
Emotion prediction accuracy across audio-recording devices

As illustrated in Fig. 8, we observed the highest predictive accuracy for the baseline recording device (*M*_Baseline_ = 51.40%), while the smartphone and lavalier achieved the lowest accuracy (*M*_Smartphone_ = 45.88% and *M*_Lavalier_ = 46.17%). As an important side finding and as illustrated in the variability of the model predictions, we observed the largest standard deviation in accuracy for the lavalier recording device (*SD*_Lavalier_ = 7.74%).

Based on this emotion classification example, we found that using amplitude and f0 measurements from different recording devices significantly impacts the predictive accuracy in an emotion prediction task, with lower accuracy for those recording devices that produce stronger deviations compared to the baseline microphone (as reported in the previous sections).

### Biological sex classification

**Fig. 9:**
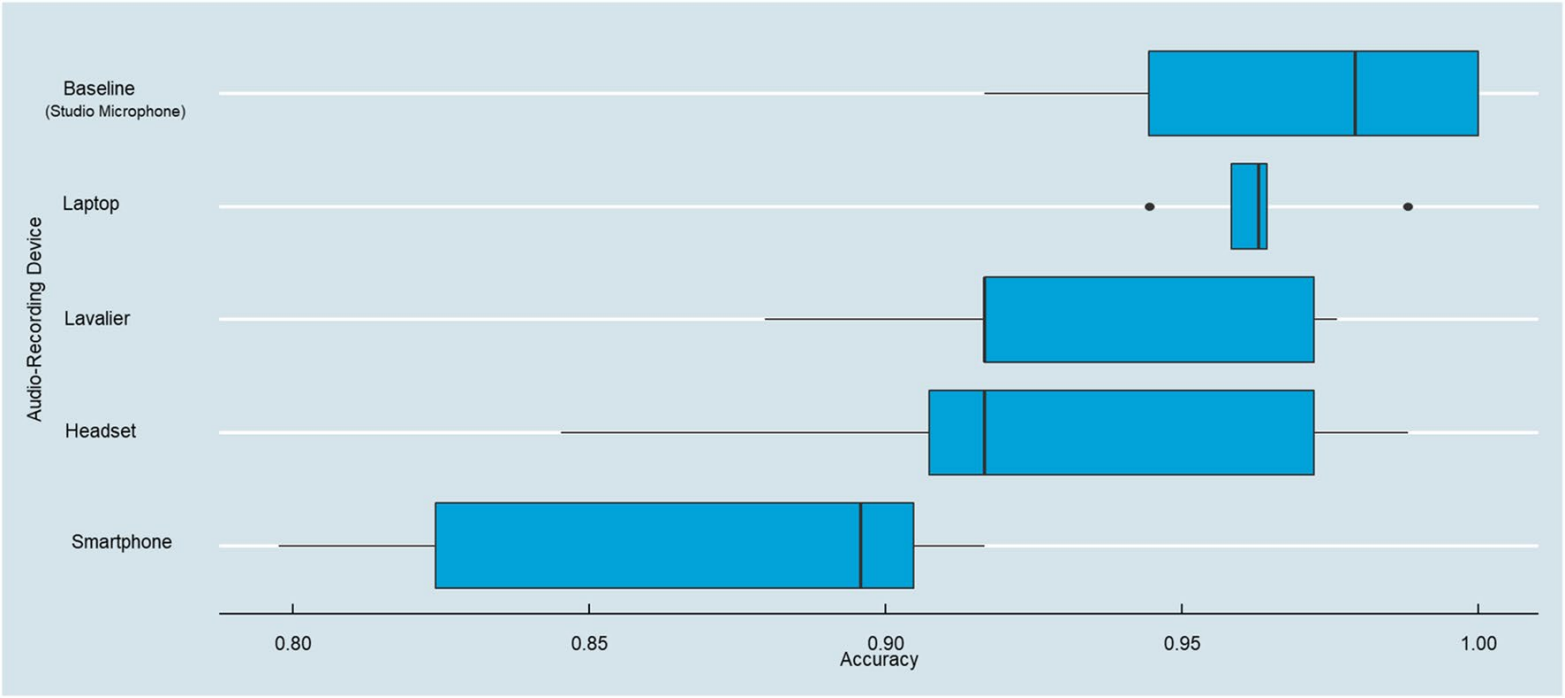
Biological sex prediction accuracy across audio-recording devices

Emotion classification is a challenging and complex classification task due to its multifaceted nature that requires a larger number of acoustic features to achieve effective classification (Anagnostopoulos et al., 2015). Critical readers may therefore argue that the previous differences in predictive accuracy are a product of such complexity. To also contrast the predictive accuracy in a setting of known high accuracy, we assessed the prediction accuracy of a participant’s biological sex. Men generally possess longer and heavier vocal folds than women, leading to lower f0 (Hillenbrand & Clark, 2009). This means that the frequency feature f0 should be a reliable and accurate predictor of biological sex, typically producing high classification rates (Bachorowski & Owren, 1999). Thus, we specified a logit model predicting male versus female participants based on their vocal features (note that all participants identified as either male or female despite the option to either prefer not to identify or identify as non-binary). As illustrated in Fig. 9, we found highly similar results for a binary classification task in which we observed the highest predictive accuracy for the baseline recording device (*M*_Baseline_ = 96.81%), while the smartphone again achieved the lowest accuracy (*M*_Smartphone_ = 86.78%). While this difference seems small, a ten-percentage point difference can evoke sizeable costs in failed speaker identification for organizations even for fairly simple tasks such as predicting the biological sex of the speaker (Bajorek, 2019). Thus, these prediction accuracy results demonstrate that the current findings are robust even in settings in which one would expect high accuracy.

### Simulation study: Robustness tests using synthetic data

Next, we conducted a simulation study to demonstrate the impact of different audio-recording devices on researchers’ substantive conclusions from voice data. To do so, we generated several synthetic datasets, each of them consisting of 480 participants, assigning each sampling unit (i.e., participant) to three different emotional states (sad, neutral, and happy—mirroring the design of our lab study) with an equal proportion of men and women in each synthetic sample, alternating the proportions of the different audio-recording devices by generating data from the recording device that overestimated amplitude (e.g., the lavalier microphone). The objective of this simulation was to rigorously assess how oversampling data from high-proximity audio-recording devices may bias substantive conclusions in a research setting.

To generate the synthetic data, we used normal probability distributions conditioned by gender, microphone type, and the emotion of the speaker. We then varied the proportion of the data stemming from one of the high-proximity devices (lavalier), equally splitting the remaining percentage among the other four devices. Specifically, we generated the following three datasets: (a) no high proximity recordings (0%) and 25% data from each of the other four devices, (b) 50% of high proximity recordings and 12.5% from the other four devices, and (c) 100% of high proximity recordings and no observations from the other four devices.

We observed a significant effect of the emotional state on the amplitude of the speakers’ voice in the condition without any high proximity device recordings (*F*(2, 1437) = 26.5, *p* < .001, η_p_^2^ = .04). Specifically, a post hoc Tukey HSD test with multiple comparison adjustment revealed a significant difference between the sad and the happy emotional states (*M*_Sad_ = 52.68 dB, *M*_Happy_ = 56.68 dB, *p* < .001) and the happy and neutral states (*M*_Neutral_ = 53.56 dB, *p* < .01), but no significant difference between the sad and the neutral states (*p* = .28).

Focusing on the sample with 50% of the observations stemming from the lavalier, and 50% from the other four devices (12.5% each), we found a significant effect of the emotional state on the amplitude of the speakers’ voice (*F*(2, 1437) = 12.43, *p* < .001, η_p_^2^ = .02). As in the previous case, we observed significant differences between the sad and the happy states (*M*_Sad_ = 57.75 dB, *M*_Happy_ = 60.72 dB, *p* < .001) and the neutral and happy states (M_Neutral_ = 58.72 dB, *p* < .01). However, no significant differences were found between the neutral and sad states (*p* = .25).

Finally, from the dataset with all observations stemming from the high-proximity lavalier audio-recording device, we observed a significant but substantially reduced effect of the emotional state on the amplitude of the speakers’ voice (*F*(2, 1437) = 4.08, *p* < .05, η_p_^2^ = .01; *M*_Sad_ = 63.17 dB, *M*_Happy_ = 64.44 dB, *p* < .05), but no significant differences between either the happy and the neutral state (*M*_Neutral_ = 63.51 dB, *p* = .11) or the sad and neutral state (*p* = .74).

**Fig. 10:**
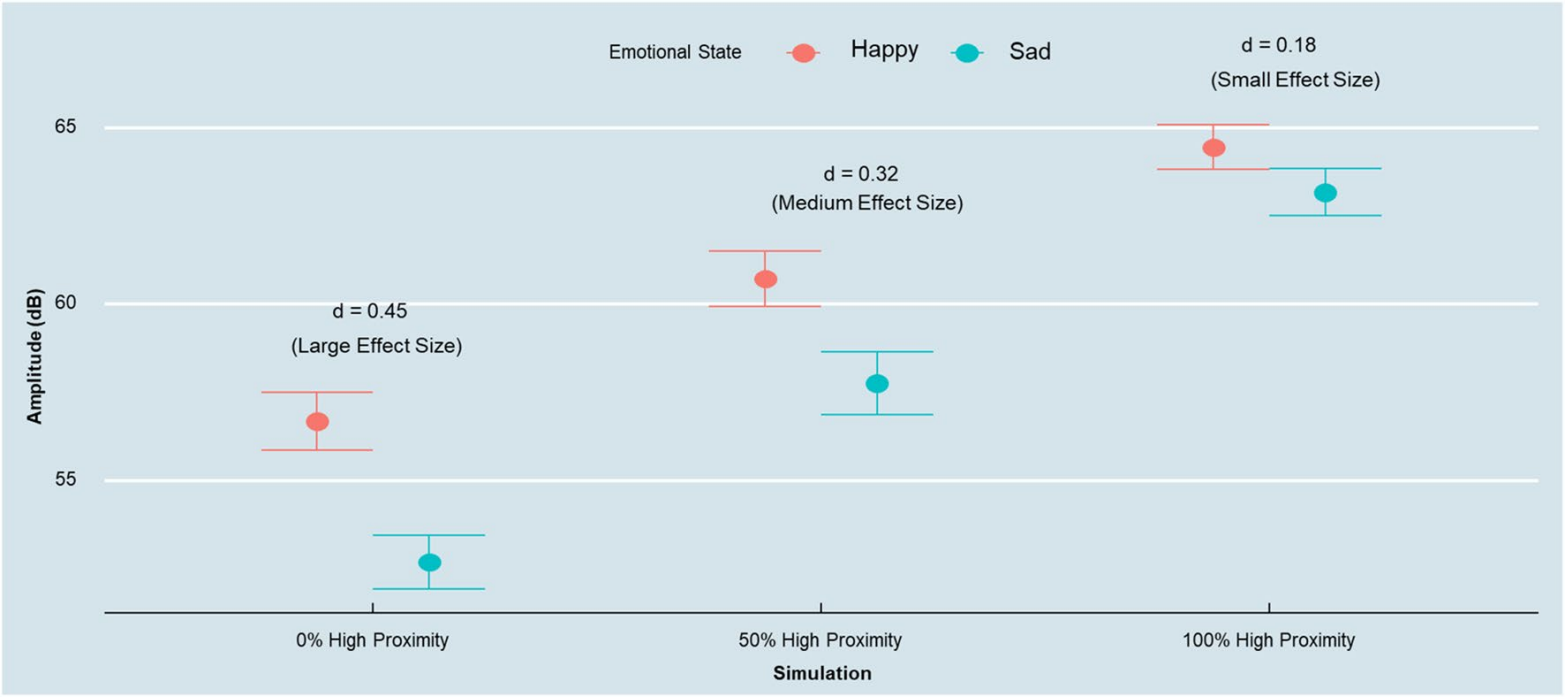
Reduced emotion detection effect sizes with an increasing share of high proximity recording devices. *Note*. *Error bars* represent a 95% confidence interval around the mean

To illustrate how these draws can impact the substantive conclusions drawn from participants’ voice samples, Fig. 10 demonstrates that while the effect sizes contrasting all three discrete emotion expression conditions are in the medium to high range in absence of high proximity devices (Cohen’s *d* = .45; average effect size based on contrasts between each discrete emotion condition), the average effect size is gradually reduced to a medium to small (*d* = .32), and ultimately small to negligible effect size (*d* = .18) for a 50% share to a full sample of high proximity devices respectively. In short, the systematically increasing amplitude of high proximity recording devices reduces any otherwise detectable condition difference in a discrete emotion expression experiment.

### A machine-learning approach to bias correction

Given the systematic differences in recording devices reported earlier, the question arises whether these biases can be corrected even after the data has been collected. While we summarize a coherent set of recording guidelines in the Discussion section, we also demonstrate one methodological approach to bias correction. Specifically, we use a machine-learning approach to bias correction with the objective to generate an objective function that can approximate the “true” underlying distribution of a vocal feature. We use the studio-recording device as a baseline and train a random forest using the vocal features (amplitude and f0) captured by the high-proximity devices as input features (lavalier and headset). We employed a random forest model due to its ability to handle non-linear relationships and the capacity to capture more complex interactions between features.

**Fig. 11:**
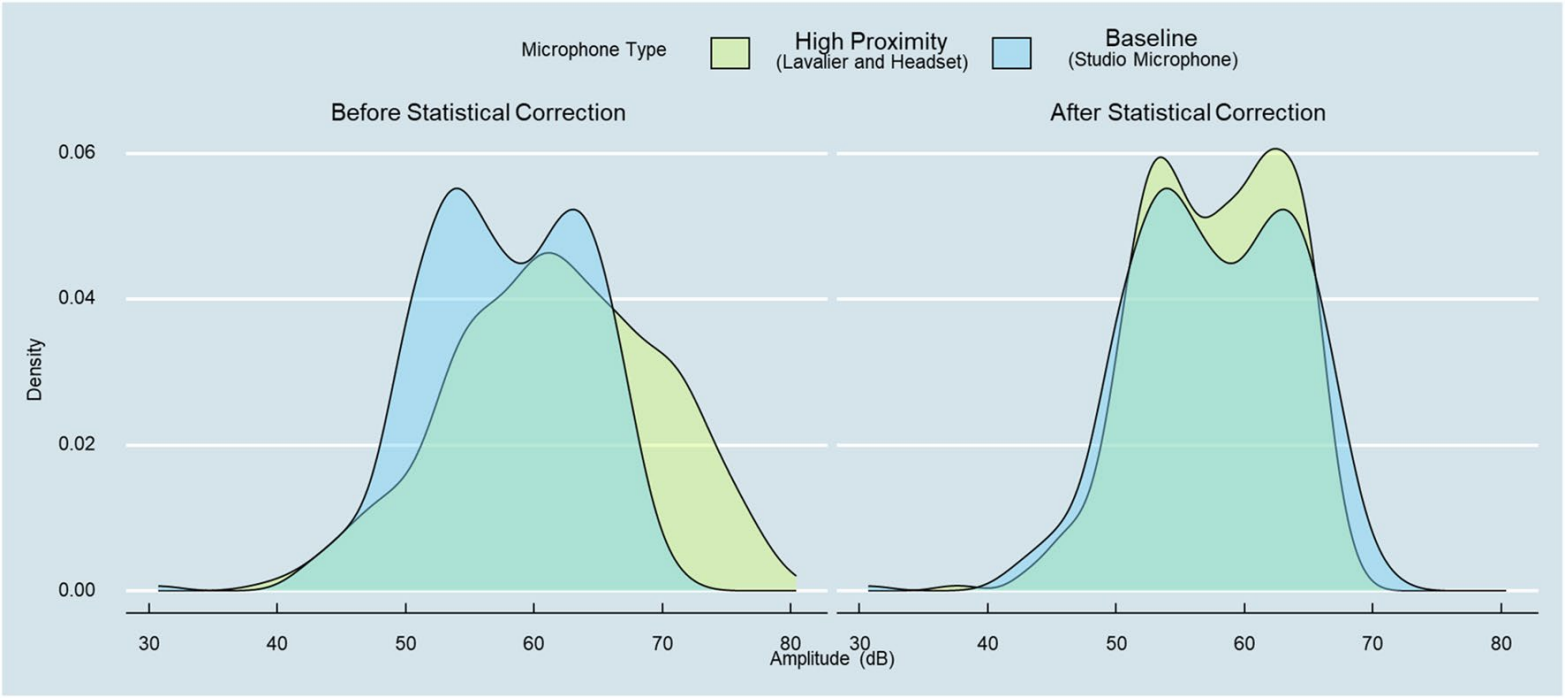
Before vs. after bias correction via random forest model

To assess the predictive accuracy of the model, we applied five-fold subject-wise cross-validation (Tougui et al., 2021). As in our preceding analyses, we demonstrate out-of-sample prediction performance to assess the ability of the model to generalize beyond the training data. Figure 11 provides a visual representation of the effectiveness of the bias correction procedure and demonstrates how the original right-skewed amplitude measure captured by the high-proximity devices is shifted toward the baseline (*R*^*2*^ = .93). These findings provide initial evidence that the distorted features of high-proximity devices were (at least somewhat) corrected by our machine-learning approach to bias correction, shifting the distribution of the upward-biased amplitude measure toward the “true” (or at least less biased) vocal features of the baseline.

We wish to highlight that this bias correction procedure requires the presence of a carefully measured baseline, which might be a luxury that is not given in every crowdsourced audio recording setting. However, we hope that it motivates researchers to first consider assessing a reasonable baseline against which the noisier data from a field setting can be calibrated against. While such bias correction procedures require more research and are beyond the scope of this paper, we hope that future work may explore this direction further.

## Discussion

An emerging line of research has started to use crowdsourced voice recordings to assess a broad range of questions, from emotion prediction using call center data to predicting a positive COVID-19 result. While facilitating large-scale and easy-to-administer data collection at low economic cost (see examples in Table 1), the question remains whether these low economic costs may come at a cost in terms of less reliable and valid data. To the best of our knowledge, no existing research we are aware of has carefully examined to which extent the same vocal features of an individual can be reliably assessed across common audio-recording devices and whether the inferences drawn are ultimately valid.

We provide causal evidence that common audio-recording devices can lead to notable differences in the vocal measurements of the human voice. Our findings highlight significant differences between recording devices to assess individuals’ f0 and amplitude from the human voice and we provide evidence that the type of device can alter researchers’ substantive conclusions: Voice recordings using a high-proximity (e.g., lavalier) microphone did not show significant differences in amplitude between happy and neutral affective states while the other devices revealed an artificial increase of the amplitude dimension. We also show that this variability between audio-recording devices influences the predictive accuracy of an emotion detection model relying on such vocal features as predictors and can cause increasingly variable prediction outcomes with a larger share of high-proximity devices (such as a lavalier microphone close to the audio source or speaker). We further show that these sizeable differences in predictive accuracy persist even for simple tasks such as predicting the biological sex of a speaker but can (to some extent) be corrected by employing a machine-learning-based bias correction procedure. Thus, our findings highlight that methodological decisions regarding audio-recording devices and recording conditions could bias conclusions drawn from audio data. For instance, a crowdsourced online experiment in which participants utilize high-proximity audio-recording devices may lead to an overestimation of amplitude measurements compared to a lab experiment in which the researcher uses consistently low-proximity devices (such as a studio-quality microphone positioned in front of a participant).

Even though our findings are relevant for any crowdsourced audio data collection where researchers have minimal control over the audio-recording devices and the recording conditions, they are also important for tightly controlled lab experiments. Envision an experiment across two labs in which recording devices are placed at varying distances from the speaker. Based on the current findings, these slight variations in the experimental setup will likely produce unreliable vocal feature measurements across recordings between labs. These findings highlight the need to provide detailed information on the research design, specific hardware setup, and to carefully monitor the recording procedures between labs. In short, the current findings have important implications for both the individual researcher interested in rigorously testing a substantive question of interest, and for future replication efforts and labs trying to replicate existing findings.

**Fig. 12:**
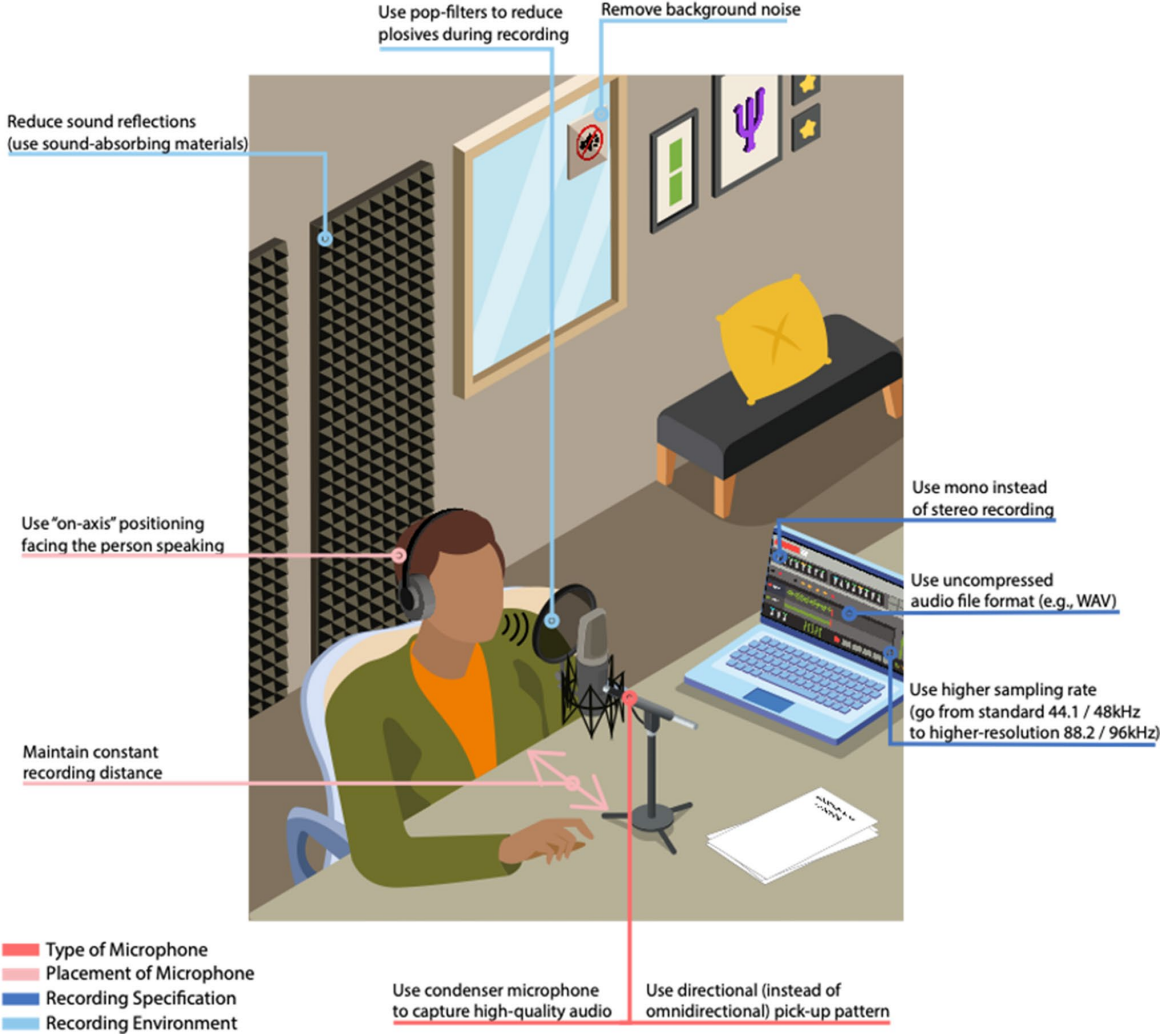
Voice recording recommendations

To provide more explicit and normative guidelines to ensure valid and reliable voice recordings, Fig. 12 provides a summary of essential guidelines and recommendations to ensure high-quality voice recordings across disciplines. We group our recommendations and guidelines into four broader areas: Type of microphone, placement of the microphone, recording specification, and recording environment. The focus of the current research has been on assessing the first two dimensions (i.e., type and placement of a microphone). Our results highlight the critical importance of using a high-quality recording device, and to avoid microphone placements that could overpower specific dimensions (such as amplitude in high-proximity devices) and recording patterns that can systematically contribute to lower-quality recordings (such as variable pick-up patterns of smartphones or laptop microphones, that typically use “omni directional” recording patterns of the microphone which can result in more unwanted background noise or other artifacts; see Web Appendix D for a summary of prior research). We also provide additional recommendations that are more universal to ensure high voice quality data (e.g., removing background noise, using sound-absorbing material, or using so-called pop filters on a microphone). For example, we recommend setting the recording setting to mono (instead of stereo) recordings to ensure a single channel for analysis, using high-quality, uncompressed audio file formats (e.g., WAV instead of MP3), and considering using a higher sampling rate. We held the recoding specification and environment factors constant in the current research and illuminated explicitly the type and placement of a microphone, but we hope that our recommendations provide helpful guidelines to ensure reliable and valid audio data collection moving forward. We also provide further details on each recommendation in the Web Appendix D.

Moving forward, we also highlight that the current findings have important implications for increasingly voice-activated technologies and firms developing user and personality profiles based on individuals’ voice recordings on these devices (such as digital voice assistants). Voice-activated devices (e.g., Amazon Alexa), smartwatches, smart soundbars, and smart TVs are all examples of devices that can capture a user’s voice data under varying recording conditions and microphones. For instance, the voice assistant Alexa Amazon Echo (2nd generation) has an array of seven microphones (Amazon.com, 2017) whereas the latest iPhone 14 has three microphones (Apple, 2022). Highlighting the importance of software innovations, the most recent Samsung smartphones (i.e., Galaxy S8 onwards) are equipped with the newly introduced feature “High Acoustic Overload Point” that improves the recording quality and filters out more aggressively background noise. Thus, voice recordings and the insights based on such unstructured forms of data are increasingly common and are likely to increase profoundly due to the increasing presence of voice-activated technologies in the marketplace (Hildebrand & Bergner, 2020; Zierau et al., 2022).

Taken together, we hope that the current findings provide fruitful directions for future research, advance current methodological practice, and highlight the critical importance of the recording device and the need for greater standardization of recording procedures. In summary, we hope that the current findings inspire future research employing voice analytics across disciplines and the critical importance of carefully describing (and documenting) the research design, hardware setup, audio processing, and analysis steps to shape the future of voice analytics across disciplines.

### Supplementary Information

Below is the link to the electronic supplementary material.

Supplementary file1 (DOCX 666 KB)

## Data Availability

All data and materials can be found in our OSF repository: https://osf.io/9g87v/?view_only=b26a8ee893f04d43a12ac965250e2438.

## Code availability

All the code is posted in our OSF repository:https://osf.io/9g87v/?view_only=b26a8ee893f04d43a12ac965250e2438.
