# Supplementary material for: Voice analytics in the wild: Validity and predictive accuracy of common audio-recording devices
Source: Behav Res Methods. 2023 May 30;56(3):2114–34. doi: 10.3758/s13428-023-02139-9 (PMC10228884; doi:10.3758/s13428-023-02139-9)
Supplement: Supplementary file 1 — Supplementary file1 (DOCX 666 KB) [file 13428_2023_2139_MOESM1_ESM.docx]

**Web Appendix**

Voice Analytics in the Wild: Validity and Predictive Accuracy of Consumer-Grade Audio-Capturing Devices

[Supplementary Analyses 2](#_Toc129474868)

[A. Results Tables 2](#_Toc129474869)

[A1. Descriptive tables – Fundamental Frequency (f0) 2](#_Toc129474870)

[A2. Descriptive tables – Amplitude 4](#_Toc129474871)

[A3. ANOVA and post-hoc contrasts tables – Fundamental Frequency 6](#_Toc129474872)

[A4. ANOVA and post-hoc contrasts tables – Amplitude 10](#_Toc129474873)

[B. Audio Features by headphones condition 14](#_Toc129474874)

[B1. Amplitude across audio-recording devices by headphones condition 14](#_Toc129474875)

[B2. Fundamental frequency across audio-recording devices by headphones condition 14](#_Toc129474876)

[Supplementary Method Information 16](#_Toc129474877)

[C. Technical details of microphones 16](#_Toc129474878)

[D. Summary of Recording Recommendations & Guidelines 17](#_Toc129474879)

# Supplementary Analyses

# Results Tables

## A1. Descriptive tables – Fundamental Frequency (f0)

Table A1.1 Means and standard deviations for f0 as a function of the recording device

| **Recording device** | *N* | *M* | *SD* |
| --- | --- | --- | --- |
| Headset | 360 | 170.88 | 26.98 |
| Lavalier | 360 | 170.86 | 26.70 |
| Baseline (Studio Microphone) | 360 | 169.31 | 26.62 |
| Smartphone | 360 | 180.70 | 36.95 |
| Laptop | 360 | 168.42 | 24.24 |

Note. *M* and *SD* represent mean and standard deviation, respectively.

Table A1.2 Means and standard deviations for f0 as a function of the recording device and the speaker's biological sex

|  | **Biological sex** | | | | | |
| --- | --- | --- | --- | --- | --- | --- |
|  | Male | | | Female | | |
| **Recording device** | *N* | *M* | *SD* | *N* | *M* | *SD* |
| Headset | 180 | 123.96 | 26.73 | 180 | 217.79 | 36.21 |
| Lavalier | 180 | 124.60 | 25.30 | 180 | 217.12 | 35.34 |
| Baseline (Studio Microphone) | 180 | 120.64 | 24.13 | 180 | 218.98 | 34.91 |
| Smartphone | 180 | 141.06 | 42.76 | 180 | 220.34 | 36.22 |
| Laptop | 180 | 119.42 | 17.84 | 180 | 217.41 | 33.31 |

Note. *M* and *SD* represent mean and standard deviation, respectively.

Table A1.3 Means and standard deviations for f0 as a function of the recording device and the speaker's simulated emotion

|  | **Simulated emotion** | | | | | | | | |
| --- | --- | --- | --- | --- | --- | --- | --- | --- | --- |
|  | Happy | | | Neutral | | | Sad | | |
| **Recording device** | *N* | *M* | *SD* | *N* | *M* | *SD* | *N* | *M* | *SD* |
| Headset | 120 | 190.56 | 24.05 | 120 | 165.35 | 17.13 | 120 | 156.73 | 18.52 |
| Lavalier | 120 | 190.76 | 23.91 | 120 | 165.93 | 17.59 | 120 | 155.69 | 15.81 |
| Baseline (Studio Microphone) | 120 | 188.37 | 26.51 | 120 | 163.74 | 16.22 | 120 | 155.82 | 15.61 |
| Smartphone | 120 | 203.35 | 32.55 | 120 | 175.50 | 29.13 | 120 | 163.26 | 26.67 |
| Laptop | 120 | 186.72 | 22.24 | 120 | 162.99 | 15.57 | 120 | 155.55 | 14.52 |

Note. *M* and *SD* represent mean and standard deviation, respectively.

Table A1.4 Means and standard deviations for f0 as a function of the recording device and participants wearing headset or not

|  | **Headset** | | | | | |
| --- | --- | --- | --- | --- | --- | --- |
|  | Present | | | Absent | | |
| **Recording device** | *N* | *M* | *SD* | *N* | *M* | *SD* |
| Headset | 180 | 169.92 | 25.81 | 180 | 171.83 | 25.81 |
| Lavalier | 180 | 169.18 | 24.75 | 180 | 172.54 | 25.53 |
| Baseline (Studio Microphone) | 180 | 167.61 | 23.42 | 180 | 171.01 | 26.62 |
| Smartphone | 180 | 180.64 | 36.25 | 180 | 180.76 | 33.47 |
| Laptop | 180 | 166.73 | 22.29 | 180 | 170.11 | 23.34 |

Note. *M* and *SD* represent mean and standard deviation, respectively.

## A2. Descriptive tables – Amplitude

Table A2.1 Means and standard deviations for amplitude as a function of the recording device

| **Recording device** | *N* | *M* | *SD* |
| --- | --- | --- | --- |
| Headset | 360 | 59.73 | 4.31 |
| Lavalier | 360 | 63.66 | 6.10 |
| Baseline (Studio Microphone) | 360 | 57.68 | 3.64 |
| Smartphone | 360 | 52.90 | 3.95 |
| Laptop | 360 | 46.91 | 4.31 |

Note. *M* and *SD* represent mean and standard deviation, respectively.

Table A2.2 Means and standard deviations for amplitude as a function of the recording device and the speaker's biological sex

|  | **Biological sex** | | | | | |
| --- | --- | --- | --- | --- | --- | --- |
|  | Male | | | Female | | |
| **Recording device** | *N* | *M* | *SD* | *N* | *M* | *SD* |
| Headset | 180 | 59.90 | 3.90 | 180 | 59.55 | 4.21 |
| Lavalier | 180 | 62.07 | 5.79 | 180 | 65.26 | 5.21 |
| Baseline (Studio Microphone) | 180 | 58.11 | 3.31 | 180 | 57.24 | 3.51 |
| Smartphone | 180 | 53.53 | 3.42 | 180 | 52.26 | 3.90 |
| Laptop | 180 | 47.26 | 4.03 | 180 | 46.55 | 4.07 |

Note. *M* and *SD* represent mean and standard deviation, respectively.

Table A2.3 Means and standard deviations for amplitude as a function of the recording device and the speaker's simulated emotion

|  | **Simulated emotion** | | | | | | | | |
| --- | --- | --- | --- | --- | --- | --- | --- | --- | --- |
|  | Happy | | | Neutral | | | Sad | | |
| **Recording device** | *N* | *M* | *SD* | *N* | *M* | *SD* | *N* | *M* | *SD* |
| Headset | 120 | 61.62 | 3.71 | 120 | 59.40 | 3.67 | 120 | 58.16 | 3.75 |
| Lavalier | 120 | 64.54 | 4.65 | 120 | 63.72 | 5.56 | 120 | 62.73 | 6.49 |
| Baseline (Studio Microphone) | 120 | 59.70 | 2.57 | 120 | 57.57 | 2.68 | 120 | 55.86 | 3.53 |
| Smartphone | 120 | 55.12 | 3.04 | 120 | 52.75 | 3.11 | 120 | 50.82 | 3.36 |
| Laptop | 120 | 48.88 | 3.74 | 120 | 46.79 | 3.54 | 120 | 45.05 | 3.67 |

Note. *M* and *SD* represent mean and standard deviation, respectively.

Table A2.4 Means and standard deviations for amplitude as a function of the recording device and speaker's headset being present or absent

|  | **Headset** | | | | | |
| --- | --- | --- | --- | --- | --- | --- |
|  | Present | | | Absent | | |
| **Recording device** | *N* | *M* | *SD* | *N* | *M* | *SD* |
| Headset | 180 | 60.00 | 4.33 | 180 | 59.46 | 3.76 |
| Lavalier | 180 | 63.09 | 6.00 | 180 | 64.24 | 5.45 |
| Baseline (Studio Microphone) | 180 | 57.09 | 3.48 | 180 | 58.27 | 3.28 |
| Smartphone | 180 | 52.40 | 3.64 | 180 | 53.39 | 3.74 |
| Laptop | 180 | 46.20 | 4.04 | 180 | 47.61 | 3.95 |

Note. *M* and *SD* represent mean and standard deviation, respectively.

## A3. ANOVA and post-hoc contrasts tables – Fundamental Frequency

Table A3.1 Results of one-way repeated-measures ANOVA of f0 by recording device

| Source | d.f. | *SS* | *MS* | *F* | *p* |
| --- | --- | --- | --- | --- | --- |
| Between groups | 4 | 2849.00 | 737.20 | 9.82 | **<.001** |
| Within groups | 116 | 8705.00 | 75.00 |  |  |
| Total | 120 | 11654.00 |  |  |  |

Note. d.f. = degrees of freedom, *SS =* sum of squares, and *MS* = mean squares

Statistically significant results are presented in bold.

Table A3.2 Post-hoc comparisons of f0 by recording device

| Comparison | Estimate | *SE* | d.f. | *t* ratio | *p* |
| --- | --- | --- | --- | --- | --- |
| Lavalier - Headset | -.02 | 2.24 | 116 | 0.01 | 1.00 |
| Lavalier - Laptop | 2.44 | 2.24 | 116 | 1.09 | .81 |
| Lavalier - Smartphone | -9.84 | 2.24 | 116 | -4.40 | **<.001** |
| Lavalier - Baseline | 1.55 | 2.24 | 116 | .69 | .96 |
| Headset - Laptop | 2.46 | 2.24 | 116 | 1.10 | .81 |
| Headset - Smartphone | -9.82 | 2.24 | 116 | -4.39 | **<.001** |
| Headset - Baseline | 1.56 | 2.24 | 116 | 0.70 | .96 |
| Laptop - Smartphone | -12.28 | 2.24 | 116 | -5.49 | **<.001** |
| Laptop - Baseline | -.89 | 2.24 | 116 | -0.40 | .99 |
| Smartphone - Baseline | 11.39 | 2.24 | 116 | 5.09 | **<.001** |

Note. *SE* and d.f. represent standard error and degrees of freedom, respectively.
Statistically significant results are presented in bold.

*p* values adjusted with Tukey HSD adjustment

Table A3.1 Results of two-way repeated-measures ANOVA of f0 by recording device and biological sex

| Source | d.f. | *SS* | *MS* | *F* | *p* |
| --- | --- | --- | --- | --- | --- |
| Biological sex | 1 | 318724 | 318724.00 | 156.80 | **<0.001** |
| Error | 28 | 56925 | 2033.00 |  |  |
| Recording device | 4 | 2949 | 737.20 | 11.83 | **<0.001** |
| Recording device x biological sex | 4 | 1724 | 431.1 | 6.92 | **<0.001** |
| Recording device x error | 112 | 6981 | 62.30 |  |  |

Note. d.f., *SS* and *MS* represent degrees of freedom, sum of squares and mean squares, respectively.

Statistically significant results are presented in bold.

Table A3.2 Post-hoc comparisons of f0 by recording device and biological sex

| Comparison | Estimate | *SE* | d.f. | *t* ratio | *p* |
| --- | --- | --- | --- | --- | --- |
| **within females** |  |  |  |  |  |
| Lavalier - Headset | -.68 | 2.88 | 112 | -.23 | 1.00 |
| Lavalier - Laptop | -.30 | 2.88 | 112 | -.10 | 1.00 |
| Lavalier - Smartphone | -3.22 | 2.88 | 112 | -1.12 | .98 |
| Lavalier - Baseline | -.86 | 2.88 | 112 | -.30 | 1.00 |
| Headset - Laptop | .38 | 2.88 | 112 | .132 | 1.00 |
| Headset - Smartphone | -.19 | 2.88 | 112 | -.06 | 1.00 |
| Headset - Baseline | -2.92 | 2.88 | 112 | -1.01 | .99 |
| Laptop - Smartphone | -.57 | 2.88 | 112 | -.20 | 1.00 |
| Laptop - Baseline | 2.35 | 2.88 | 112 | .82 | 1.00 |
| Smartphone - Baseline | -.68 | 2.88 | 112 | -.23 | 1.00 |
| **within males** |  |  |  |  |  |
| Lavalier - Headset | .64 | 2.88 | 112 | .22 | 1.00 |
| Lavalier - Laptop | 5.18 | 2.88 | 112 | 1.80 | .74 |
| Lavalier - Smartphone | -16.46 | 2.88 | 112 | -5.71 | **<.001** |
| Lavalier - Baseline | 3.96 | 2.88 | 112 | 1.37 | .93 |
| Headset - Laptop | 4.54 | 2.88 | 112 | 1.57 | .86 |
| Headset - Smartphone | -17.11 | 2.88 | 112 | -5.93 | **<.001** |
| Headset - Baseline | 3.32 | 2.88 | 112 | 1.15 | .98 |
| Laptop - Smartphone | -21.64 | 2.88 | 112 | -7.51 | **<.001** |
| Laptop - Baseline | -1.22 | 2.88 | 112 | -.42 | 1.00 |
| Smartphone - Baseline | 20.42 | 2.88 | 112 | 7.08 | **<.001** |

Note. *SE* and d.f. represent standard error and degrees of freedom, respectively.
Statistically significant results are presented in bold.

*p* values adjusted with Tukey HSD adjustment

Table A3.5 Results of two-way repeated-measures ANOVA of f0 by recording device and simulated emotion

| Source | d.f. | *SS* | *MS* | *F* | *p* |
| --- | --- | --- | --- | --- | --- |
| Simulated emotion | 2 | 897.50 | 448.70 | 28.77 | **<0.001** |
| Error | 58 | 904.50 | 15.60 |  |  |
| Recording device | 4 | 15088 | 3772 | 148.70 | **<0.001** |
| Recording device x simulated emotion | 8 | 59.57 | 7.45 | 16.00 | **<0.001** |
| Recording device x error | 232 | 107.93 | 0.46 |  |  |

Note. d.f., *SS* and *MS* represent degrees of freedom, sum of squares and mean squares, respectively.

Statistically significant results are presented in bold.

Table A3.6 Post-hoc comparisons of f0 by recording device and simulated emotion

| Comparison | Estimate | *SE* | d.f. | *t* ratio | *p* |
| --- | --- | --- | --- | --- | --- |
| **within happy** |  |  |  |  |  |
| Lavalier - Headset | .41 | 2.60 | 198.90 | .16 | 1.00 |
| Lavalier - Laptop | 4.25 | 2.60 | 198.90 | 1.64 | .95 |
| Lavalier - Smartphone | -12.38 | 2.60 | 198.90 | -4.77 | **<.001** |
| Lavalier - Baseline | 2.59 | 2.60 | 198.90 | 1.00 | 1.00 |
| Headset - Laptop | 3.84 | 2.60 | 198.90 | 1.48 | .98 |
| Headset - Smartphone | -12.79 | 2.60 | 198.90 | -4.92 | .00 |
| Headset - Baseline | 2.18 | 2.60 | 198.90 | .84 | 1.00 |
| Laptop - Smartphone | -16.63 | 2.60 | 198.90 | -6.40 | **<.001** |
| Laptop - Baseline | -1.66 | 2.60 | 198.90 | -.64 | 1.00 |
| Smartphone - Baseline | 14.97 | 2.60 | 198.90 | 5.76 | **<.001** |
| **within neutral** |  |  |  |  |  |
| Lavalier - Headset | .58 | 2.60 | 198.90 | .22 | 1.00 |
| Lavalier - Laptop | 2.94 | 2.60 | 198.90 | 1.13 | 1.00 |
| Lavalier - Smartphone | -9.57 | 2.60 | 198.90 | -3.68 | **.02** |
| Lavalier - Baseline | 2.19 | 2.60 | 198.90 | .84 | 1.00 |
| Headset - Laptop | 2.35 | 2.60 | 198.90 | .91 | 1.00 |
| Headset - Smartphone | 1.60 | 2.60 | 198.90 | .62 | 1.00 |
| Headset - Baseline | -12.51 | 2.60 | 198.90 | -4.81 | **<.001** |
| Laptop - Smartphone | -.75 | 2.60 | 198.90 | -.29 | 1.00 |
| Laptop - Baseline | 11.75 | 2.60 | 198.90 | 4.53 | **<.001** |
| Smartphone - Baseline | .58 | 2.60 | 198.90 | .22 | 1.00 |
| **within sad** |  |  |  |  |  |
| Lavalier - Headset | -1.04 | 2.60 | 198.90 | -.40 | 1.00 |
| Lavalier - Laptop | .14 | 2.60 | 198.90 | .05 | 1.00 |
| Lavalier - Smartphone | -7.57 | 2.60 | 198.90 | -2.92 | .20 |
| Lavalier - Baseline | -.13 | 2.60 | 198.90 | -.05 | 1.00 |
| Headset - Laptop | 1.18 | 2.60 | 198.90 | .45 | 1.00 |
| Headset - Smartphone | -6.53 | 2.60 | 198.90 | -2.51 | .44 |
| Headset - Baseline | .91 | 2.60 | 198.90 | .35 | 1.00 |
| Laptop - Smartphone | -7.71 | 2.60 | 198.90 | -2.97 | .18 |
| Laptop - Baseline | -.27 | 2.60 | 198.90 | -.10 | 1.00 |
| Smartphone - Baseline | 7.44 | 2.60 | 198.90 | 2.86 | .22 |

Note. *SE* and d.f. represent standard error and degrees of freedom, respectively.
Statistically significant results are presented in bold.

*p* values adjusted with Tukey HSD adjustment

Table A3.7 Results of two-way repeated-measures ANOVA of f0 by recording device and participant wearing headphones or not

| Source | d.f. | *SS* | *MS* | *F* | *p* |
| --- | --- | --- | --- | --- | --- |
| Headphones | 1 | 444 | 444.30 | 2.13 | .16 |
| Error | 29 | 751299 | 25907.00 |  |  |
| Recording device | 4 | 5898 | 1474.40 | 9.83 | **<.001** |
| Recording device x headphones | 4 | 125 | 31.24 | 1.04 | .39 |
| Recording device x error | 116 | 3495 | 30.13 |  |  |

Note. d.f., *SS* and *MS* represent degrees of freedom, sum of squares and mean squares, respectively. Statistically significant results are presented in bold.

## A4. ANOVA and post-hoc contrasts tables – Amplitude

Table A4.1 Results of one-way repeated-measures ANOVA of amplitude by recording device

| Source | d.f. | *SS* | *MS* | *F* | *p* |
| --- | --- | --- | --- | --- | --- |
| Error | 29 | 6196 | 213.70 |  |  |
| Recording device | 4 | 5029 | 1257.30 | 148.70 | **<0.001** |
| Recording device x error | 116 | 981 | 8.50 |  |  |

Note. d.f., *SS* and *MS* represent degrees of freedom, sum of squares and mean squares, respectively.

Statistically significant results are presented in bold.

Table A4.2 Post-hoc comparisons of amplitude between recording device

| Comparison | Estimate | *SE* | d.f. | *t* ratio | *p* |
| --- | --- | --- | --- | --- | --- |
| Lavalier - Headset | 3.94 | .75 | 116 | 5.24 | **<.001** |
| Lavalier - Laptop | 16.76 | .75 | 116 | 22.32 | **<.001** |
| Lavalier - Smartphone | 10.77 | .75 | 116 | 14.34 | **<.001** |
| Lavalier - Baseline | 5.99 | .75 | 116 | 7.98 | **<.001** |
| Headset - Laptop | 12.82 | .75 | 116 | 17.08 | **<.001** |
| Headset - Smartphone | 6.83 | .75 | 116 | 9.10 | **<.001** |
| Headset - Baseline | 2.05 | .75 | 116 | 2.73 | .06 |
| Laptop - Smartphone | -5.99 | .75 | 116 | -7.98 | **<.001** |
| Laptop - Baseline | -10.77 | .75 | 116 | -14.34 | **<.001** |
| Smartphone - Baseline | -4.78 | .75 | 116 | -6.37 | **<.001** |

Note. *SE* and d.f. represent standard error and degrees of freedom, respectively.
Statistically significant results are presented in bold.

*p* values adjusted with Tukey HSD adjustment

Table A4.3 Results of two-way repeated-measures ANOVA of amplitude by recording device and biological sex

| Source | d.f. | *SS* | *MS* | *F* | *p* |
| --- | --- | --- | --- | --- | --- |
| Biological sex | 1 | 19 | 19.30 | .09 | .77 |
| Error | 28 | 6177 | 220.60 |  |  |
| Recording device | 4 | 5029 | 1257.30 | 159.61 | **<.001** |
| Recording device x biological sex | 4 | 99 | 24.70 | 3.14 | **.02** |
| Recording device x error | 112 | 882 | 7.90 |  |  |

Note. d.f., *SS* and *MS* represent degrees of freedom, sum of squares and mean squares, respectively.

Statistically significant results are presented in bold.

Table A4.4 Post-hoc comparisons of amplitude by recording device and biological sex

| Comparison | Estimate | *SE* | d.f. | *t* ratio | *p* |
| --- | --- | --- | --- | --- | --- |
| **within females** |  |  |  |  |  |
| Lavalier - Headset | 5.71 | 1.02 | 112.00 | 5.57 | **<.001** |
| Lavalier - Laptop | 18.71 | 1.02 | 112.00 | 18.26 | **<.001** |
| Lavalier - Smartphone | 13.00 | 1.02 | 112.00 | 12.69 | **<.001** |
| Lavalier - Baseline | 8.02 | 1.02 | 112.00 | 7.82 | **<.001** |
| Headset - Laptop | 13.00 | 1.02 | 112.00 | 12.69 | **<.001** |
| Headset - Smartphone | 7.29 | 1.02 | 112.00 | 7.12 | **<.001** |
| Headset - Baseline | 2.31 | 1.02 | 112.00 | 2.26 | .43 |
| Laptop - Smartphone | -5.71 | 1.02 | 112.00 | -5.57 | **<.001** |
| Laptop - Baseline | -10.69 | 1.02 | 112.00 | -10.43 | **<.001** |
| Smartphone - Baseline | -4.98 | 1.02 | 112.00 | -4.86 | **<0.001** |
| **within males** |  |  |  |  |  |
| Lavalier - Headset | 2.16 | 1.02 | 112.00 | 2.11 | .52 |
| Lavalier - Laptop | 14.81 | 1.02 | 112.00 | 14.45 | **<.001** |
| Lavalier - Smartphone | 8.54 | 1.02 | 112.00 | 8.33 | **<.001** |
| Lavalier - Baseline | 3.96 | 1.02 | 112.00 | 3.86 | **.01** |
| Headset - Laptop | 12.64 | 1.02 | 112.00 | 12.34 | **<.001** |
| Headset - Smartphone | 6.37 | 1.02 | 112.00 | 6.22 | **<.001** |
| Headset - Baseline | 1.80 | 1.02 | 112.00 | 1.75 | .76 |
| Laptop - Smartphone | -6.27 | 1.02 | 112.00 | -6.12 | **<.001** |
| Laptop - Baseline | -10.85 | 1.02 | 112.00 | -10.59 | **<.001** |
| Smartphone - Baseline | -4.58 | 1.02 | 112.00 | -4.47 | **.001** |

Note. *SE* and d.f. represent standard error and degrees of freedom, respectively.
Statistically significant results are presented in bold.

*p* values adjusted with Tukey HSD adjustment

Table A4.5 Results of two-way repeated-measures ANOVA of amplitude by recording device and simulated emotion

| Source | d.f. | *SS* | *MS* | *F* | *p* |
| --- | --- | --- | --- | --- | --- |
| Simulated emotion | 2 | 897.50 | 448.70 | 28.77 | **<.001** |
| Error | 58 | 904.50 | 15.60 |  |  |
| Recording device | 4 | 15088 | 3772.00 | 148.70 | **<.001** |
| Recording device x simulated emotion | 8 | 59.57 | 7.45 | 16.00 | **<.001** |
| Recording device x error | 232 | 107.93 | 0.47 |  |  |

Note. d.f., *SS* and *MS* represent degrees of freedom, sum of squares and mean squares, respectively.

Statistically significant results are presented in bold.

Table A4.6 Post-hoc comparisons of amplitude by recording device and simulated emotion

| Comparison | Estimate | *SE* | d.f. | *t* ratio | *p* |
| --- | --- | --- | --- | --- | --- |
| **within happy** |  |  |  |  |  |
| Lavalier - Headset | 2.92 | .77 | 124.60 | 3.82 | **.02** |
| Lavalier - Laptop | 15.66 | .77 | 124.60 | 20.49 | **<.001** |
| Lavalier - Smartphone | 9.42 | .77 | 124.60 | 12.32 | **<.001** |
| Lavalier - Baseline | 4.84 | .77 | 124.60 | 6.33 | **<.001** |
| Headset - Laptop | 12.74 | .77 | 124.60 | 16.66 | **<.001** |
| Headset - Smartphone | 6.50 | .77 | 124.60 | 8.50 | **<.001** |
| Headset - Baseline | 1.92 | .77 | 124.60 | 2.51 | .44 |
| Laptop - Smartphone | -6.24 | .77 | 124.60 | -8.17 | **<.001** |
| Laptop - Baseline | -10.82 | .77 | 124.60 | -14.16 | **<.001** |
| Smartphone - Baseline | -4.58 | .77 | 124.60 | -5.99 | **<.001** |
| **within neutral** |  |  |  |  |  |
| Lavalier - Headset | 4.31 | .77 | 124.60 | 5.64 | **<.001** |
| Lavalier - Laptop | 16.93 | .77 | 124.60 | 22.14 | **<.001** |
| Lavalier - Smartphone | 10.97 | .77 | 124.60 | 14.35 | **<.001** |
| Lavalier - Baseline | 6.25 | .77 | 124.60 | 8.18 | **<.001** |
| Headset - Laptop | 12.61 | .77 | 124.60 | 16.50 | **<.001** |
| Headset - Smartphone | 1.94 | .77 | 124.60 | 2.53 | .43 |
| Headset - Baseline | -5.95 | .77 | 124.60 | -7.79 | **<.001** |
| Laptop - Smartphone | -10.68 | .77 | 124.60 | -13.96 | **<.001** |
| Laptop - Baseline | -4.72 | .77 | 124.60 | -6.17 | **<.001** |
| Smartphone - Baseline | 4.31 | .77 | 124.60 | 5.64 | **<.001** |
| **within sad** |  |  |  |  |  |
| Lavalier - Headset | 4.57 | .77 | 124.60 | 5.98 | **<.001** |
| Lavalier - Laptop | 17.69 | .77 | 124.60 | 23.14 | **<.001** |
| Lavalier - Smartphone | 11.92 | .77 | 124.60 | 15.59 | **<.001** |
| Lavalier - Baseline | 6.88 | .77 | 124.60 | 9.00 | **<.001** |
| Headset - Laptop | 13.12 | .77 | 124.60 | 17.16 | **<.001** |
| Headset - Smartphone | 7.35 | .77 | 124.60 | 9.61 | **<.001** |
| Headset - Baseline | 2.31 | .77 | 124.60 | 3.02 | .16 |
| Laptop - Smartphone | -5.77 | .77 | 124.60 | -7.55 | **<.001** |
| Laptop - Baseline | -10.81 | .77 | 124.60 | -14.14 | **<.001** |
| Smartphone - Baseline | -5.04 | .77 | 124.60 | -6.59 | **<.001** |

Note. *SE* and d.f. represent standard error and degrees of freedom, respectively.
Statistically significant results are presented in bold.

*p* values adjusted with Tukey HSD adjustment

Table A4.7 Results of two-way repeated-measures ANOVA of amplitude by recording device and participant wearing headphones or not

| Source | d.f. | *SS* | *MS* | *F* | *p* |
| --- | --- | --- | --- | --- | --- |
| Headphones | 1 | 52.39 | 7.96 | 7.96 | **.008** |
| Error | 29 | 12393 | 427.30 |  |  |
| Recording device | 4 | 10058 | 2514.60 | 148.70 | **<.001** |
| Recording device x headphones | 4 | 37.06 | 9.27 | 29.65 | **<.001** |
| Recording device x error | 116 | 36.24 | .31 |  |  |

Note. d.f., *SS* and *MS* represent degrees of freedom, sum of squares and mean squares, respectively.

Statistically significant results are presented in bold.

Table A4.8 Post-hoc comparisons of amplitude by recording device and participant wearing headphones or not

| Comparison | Estimate | *SE* | d.f. | *t* ratio | *p* |
| --- | --- | --- | --- | --- | --- |
| **within participants wearing headphones** |  |  |  |  |  |
| Lavalier - Headset | 3.09 | .76 | 120.30 | 4.08 | **.003** |
| Lavalier - Laptop | 16.89 | .76 | 120.30 | 22.29 | **<.001** |
| Lavalier - Smartphone | 10.69 | .76 | 120.30 | 14.11 | **<.001** |
| Lavalier - Baseline | 6.01 | .76 | 120.30 | 7.93 | **<.001** |
| Headset - Laptop | 13.80 | .76 | 120.30 | 18.21 | **<.001** |
| Headset - Smartphone | 7.60 | .76 | 120.30 | 10.03 | **<.001** |
| Headset - Baseline | 2.92 | .76 | 120.30 | 3.85 | **.007** |
| Laptop - Smartphone | -6.20 | .76 | 120.30 | -8.18 | **<.001** |
| Laptop - Baseline | -10.88 | .76 | 120.30 | -14.36 | **<.001** |
| Smartphone - Baseline | -4.68 | .76 | 120.30 | -6.18 | **<.001** |
| **within participants not wearing headphones** |  |  |  |  |  |
| Lavalier - Headset | 4.78 | .76 | 120.30 | 6.31 | **<.001** |
| Lavalier - Laptop | 16.63 | .76 | 120.30 | 21.94 | **<.001** |
| Lavalier - Smartphone | 10.85 | .76 | 120.30 | 14.32 | **<.001** |
| Lavalier - Baseline | 5.97 | .76 | 120.30 | 7.88 | **<.001** |
| Headset - Laptop | 11.85 | .76 | 120.30 | 15.63 | **<.001** |
| Headset - Smartphone | 1.19 | .76 | 120.30 | 1.57 | .86 |
| Headset - Baseline | -5.78 | .76 | 120.30 | -7.63 | **<.001** |
| Laptop - Smartphone | -10.65 | .76 | 120.30 | -14.06 | **<.001** |
| Laptop - Baseline | -4.88 | .76 | 120.30 | -6.43 | **<.001** |
| Smartphone - Baseline | 4.78 | .76 | 120.30 | 6.31 | **<.001** |

Note. *SE* and d.f. represent standard error and degrees of freedom, respectively.
Statistically significant results are presented in bold.

*p* values adjusted with Tukey HSD adjustment

# Audio Features by headphones condition

## B1. Amplitude across audio-recording devices by headphones condition

We assessed whether the systematic differences in amplitude across audio-recoding devices vary between participants wearing headphones and not wearing headphones. Overall, the absolute differences between recording devices remained robust and in the same direction as reported in section 4.1 of the main manuscript. As an exploratory side result, we found a significant interaction effect between the audio-recording device and the headphones condition (*F*(4, 116) = 29.65, *p* < .001, η_p_^2^ = .51). Probing the interaction revealed that, for participants wearing headphones, all devices presented significant differences in amplitude across them (*M*_Headset_ = 60.00, *M*_Lavalier_ = 63.09, *M*_Baseline_ = 57.09, *M*_Smartphone_ = 52.40, *M*_Laptop_ = 46.20, all *p* < .01). However, for participants not wearing headphones, the headset and the baseline microphone did not present a significant difference in amplitude (*M*_Headset_ = 59.46, *M*_Baseline_ = 58.28, *t* = 1.57, *p* = .86).

Fig. 3 Amplitude across audio-recording devices by headphones condition.


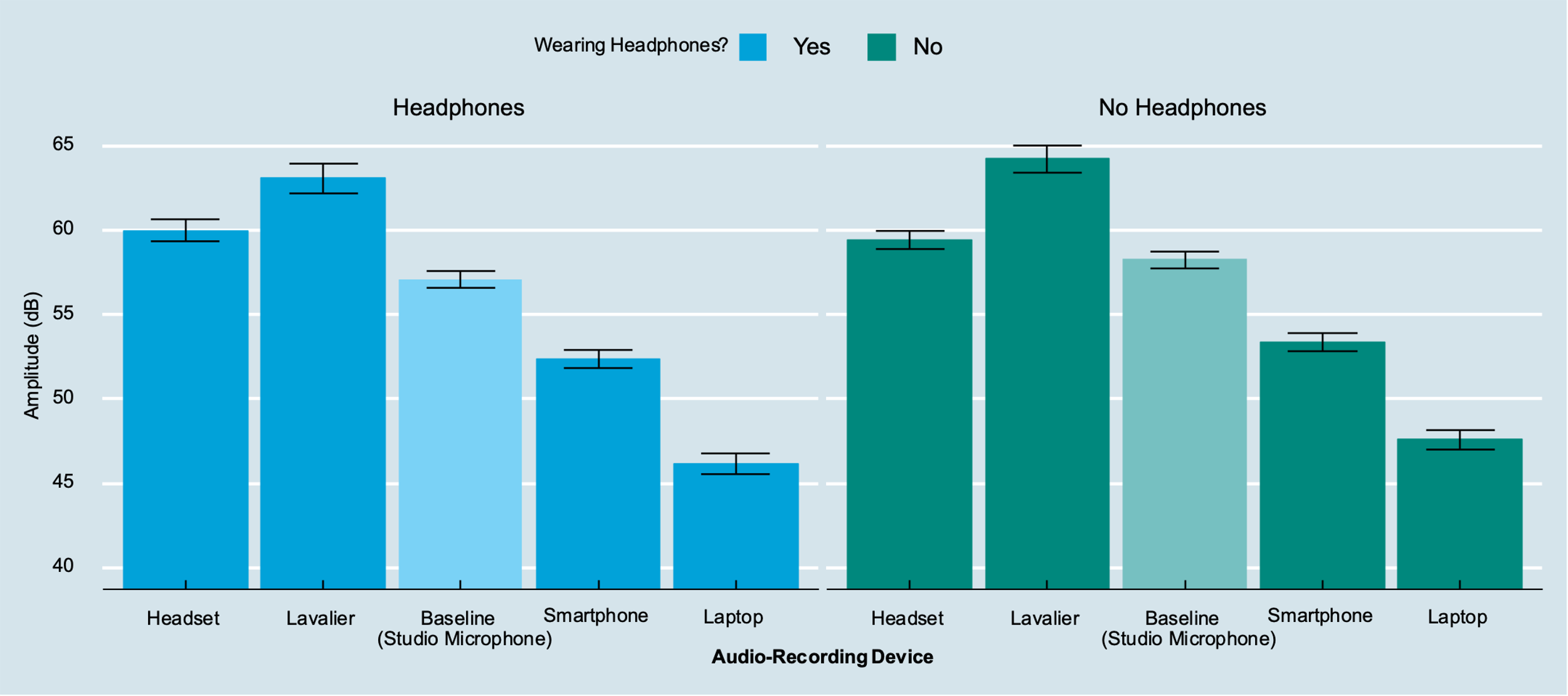


*Note*: Error bars represent 95% confidence interval around the mean.

## B2. Fundamental frequency across audio-recording devices by headphones condition

We assessed whether the differences across audio-recording devices vary when participants wear headphones or not, we did not observe a significant interaction effect between audio-recording devices and headphones (*F*(4, 116) = 1.04, *p* = .39, η_p_^2^ = .03).

Fig. B2.1 Fundamental frequency across audio-capturing devices by headphones condition.


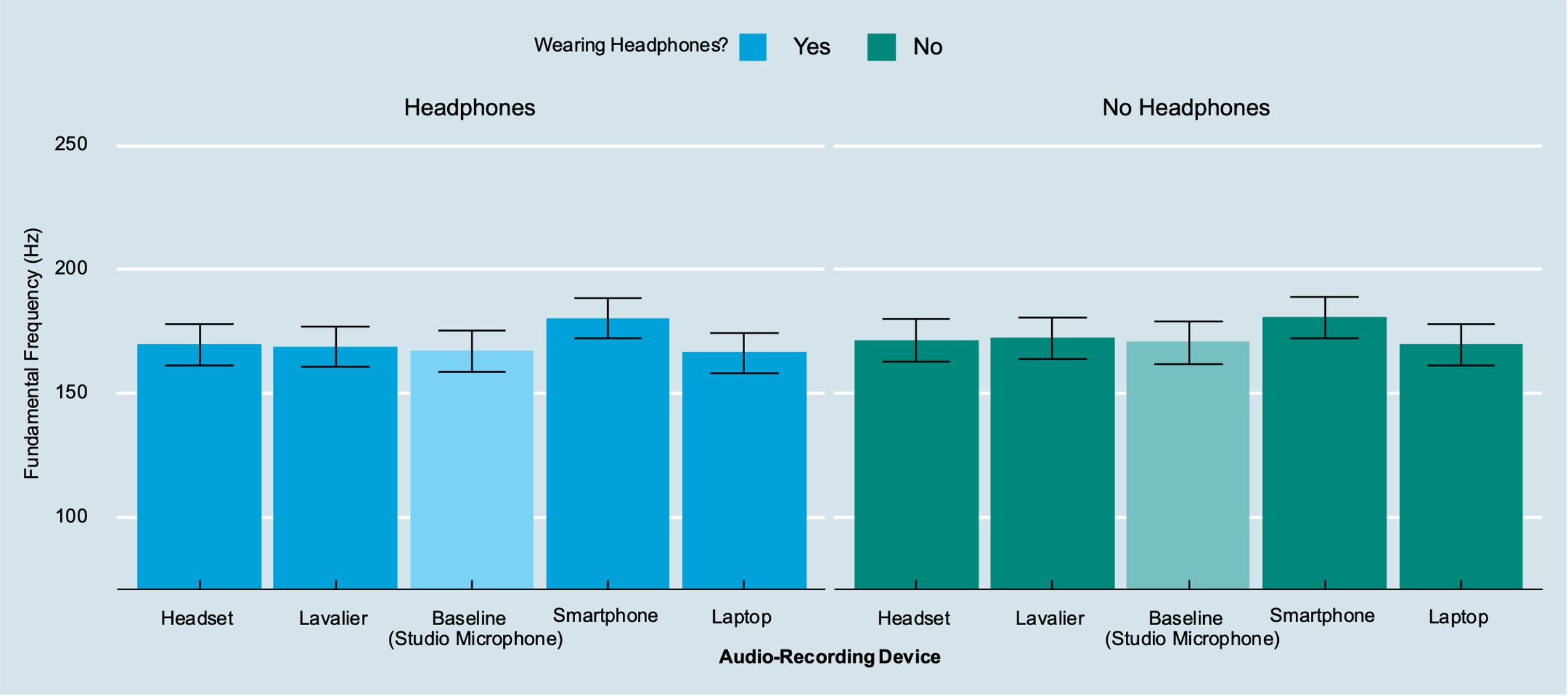


*Note*: Error bars represent 95% confidence interval around the mean.

# Supplementary Method Information

# Technical details of microphones

Prior work on the technical aspects of microphones and audio recording devices suggests that the frequency range, the dynamic range, and the polar pattern of a microphone are important for accurate measurements of speech (Svec 2010). These details are often not explicitly reported in consumer-grade recording devices (as shown in Table C1 and discussed in the main manuscript of the current research).

Table C1 Technical specifications of the microphones tested in the setting of this paper.

| Audio-recording device | Frequency Range | Dynamic Range | Polar  Pattern | Additional Filters |
| --- | --- | --- | --- | --- |
| Lavalier | 20 Hz – 20 kHz | 83 dB | Omnidirectional | Noise protection filter |
| Headset | Not reported | Not reported | Not reported | Not reported |
| Studio (Baseline) | 20 Hz – 20 kHz | 114 dB | Cardioid / Omnidirectional /  Bi-directional /  Stereo  (In our setting we used Cardioid) | No |
| Smartphone | Not reported | Not reported | Not reported | Not reported |
| Laptop | Not reported | Not reported | Directional beamforming | Three-microphone array |

# Summary of Recording Recommendations & Guidelines

|  | Characteristic | Description | Recommendation | Related Work |
| --- | --- | --- | --- | --- |
| Recording  Environment |  |  |  |  |
|  | Background noise | Background noise refers to any unwanted sounds in the environment that could negatively impact the recording quality. | Avoid background noise whenever possible. Avoid any interfering disturbance of the captured audio signal by ensuring a quiet recording environment without any additional, unwanted sound sources (such as air-conditioners, fans, or open windows). Directional microphones can help reduce the impact of background noise. | Vogel & Morgan, 2009; Parsa & Jamieson, 2001 |
|  | Echo / Sound reflections | Echo is the reflection of sound waves based on the surrounding surfaces of the recording setting. | Ensure, whenever possible, sound-absorbing materials in the recording environment to reduce the amount of reflected sound and to record in smaller rooms. Researchers can capture room size as control variables and (if possible) ask participants to cover surrounding surfaces with available, sound-absorbing materials (such as using blankets or drawing the curtains in front of a window). | Vogel & Morgan, 2009 |
|  | Pop filters | Microphone filters are external devices that reduce or eliminate unwanted sounds from an audio signal. | Pop filters are effective external devices that reduce the blast of plosive energy from a speaker. These physical add-ons greatly enhance the quality of the voice recording when recording in a direct, on-axis recording setting. | Salih, 2017; Shiota et al., 2015 |
| Type of Microphone |  |  |  |  |
|  | Directionality | Microphone directionality refers to the sensitivity of microphones to capture sounds coming from different directions. | Researchers should ask for a specific directionality pattern (if possible, adjusting it), or control for the type of microphone that is being used. Omnidirectional microphones are more prone to record background noise or unwanted sound signals in the environment. | Švec & Granqvist, 2010; Titze & Winholtz, 1993; Parsa & Jamieson, 2001 |
|  | Transducer type | A transducer is the part of the microphone that transforms sound waves into electrical impulses. Dynamic and condenser transducers are the most common. | Condenser microphones generally produce better recording quality of voice data compared to dynamic microphones. Use condenser microphones if possible or statistically control for the type of transducer microphone. | Švec & Granqvist, 2010; Titze & Winholtz, 1993; Parsa & Jamieson, 2001 |
| Placement of Microphone |  |  |  |  |
|  | Distance | The spatial distance between the microphone and sound source (e.g., speaker). | The distance between the microphone and the speaker varies as a function of the recording device (e.g., headset microphones are typically closer to the speaker than built-in laptop or tablet microphones). Researchers should instruct participants to maintain a constant distance throughout the recording. We also advice to at least statistically control for the type of recording device. | Švec & Granqvist, 2010; Titze & Winholtz, 1993; Parsa & Jamieson, 2001; This paper |
|  | Angle | The angle refers to the direction of the soundwave from the speaker’s mouth to the microphone. | The optimal angle to record audio varies depending on the type of microphone and the distance between the speaker and microphone. In most cases, we recommend an “on-axis” recording in which the microphone is directly facing the person speaking. While this on-axis recording can induce so-called “plosives” (i.e., breath-heavy consonants such as “p” or “t” that interrupt or induce noisy sounds during the recording). Researchers should therefore at least instruct participants to use a consistent, direct angle facing the microphone. | Švec & Granqvist, 2010; Titze & Winholtz, 1993; Parsa & Jamieson, 2001 |
|  | Pop filters | Microphone filters are external devices that reduce or eliminate unwanted sounds from an audio signal. | Pop filters are effective external devices that reduce the blast of plosive energy from a speaker. These physical add-ons greatly enhance the quality of the voice recording when recording in a direct, on-axis recording setting. | Salih, 2017; Shiota et al., 2015 |
| Recording Specification |  |  |  |  |
|  | Mono vs. Stereo | Mono records a single channel whereas stereo records two or more channels. | While most recording software applications record in stereo by default (i.e., two channels), voice recordings of a single individual should generally be recorded in mono (i.e., one channel) to avoid picking up unwanted additional sound sources. | Eargle, 2012 |
|  | Audio format | Audio format refers to the digital representation of a recorded sound. Common audio formats include WAV and MP3, with the former being lossless and not compressed (i.e., higher recording quality). | In general, use uncompressed WAV format if possible to avoid compression-related quality loss. Alternatively, researchers can request participants to submit a sample recording to ensure that the audio format is set correctly. | Siegert et al., 2016 |
|  | Sampling rate | The sampling rate captures the number of samples per second of a soundwave. The more samples per second, the higher the quality of the recording. | Researchers should provide clear instructions on the required sampling rate and guidelines on how to set it up in the recording software. Additionally, researchers can request participants to submit a sample recording. | Stiros et al., 2008 |

References

Eargle, J. (2012). *The Microphone Book: From mono to stereo to surround. A guide to microphone design and application*. Routledge.

Salih, A. O. M. (2017). Audio noise reduction using low-pass filters. *Open Access Library Journal*, *4*(11), 1–7.

Shiota, S., Villavicencio, F., Yamagishi, J., Ono, N., Echizen, I., & Matsui, T. (2015). Voice liveness detection algorithms based on pop noise caused by human breath for automatic speaker verification. *Sixteenth Annual Conference of the International Speech Communication Association*.

Siegert, I., Lotz, A. F., Duong, L. L., & Wendemuth, A. (2016). Measuring the impact of audio compression on the spectral quality of speech data. *Studientexte Zur Sprachkommunikation: Elektronische Sprachsignalverarbeitung 2016*, 229–236.

Stiros, S., Psimoulis, P., & Kokkinou, E. (2008). Errors introduced by fluctuations in the sampling rate of automatically recording instruments: Experimental and theoretical approach. *Journal of Surveying Engineering*, *134*(3), 89–93.

Švec, J. G., & Granqvist, S. (2010). Guidelines for selecting microphones for human voice production research. *American Journal of Speech-Language Pathology*, *19*(4), 356–368. https://doi.org/10.1044/1058-0360(2010/09-0091)
